# Supplementary material for: Performance evaluation of inclusive green growth in China: Dynamic evolution, regional differences, and spatial correlation
Source: PLoS One. 2024 Jul 25;19(7):e0305338. doi: 10.1371/journal.pone.0305338 (PMC11271904; doi:10.1371/journal.pone.0305338)
Supplement: S1 Appendix — (DOCX) [file pone.0305338.s001.docx]

The data for Fig 1

| year | Q1 | Q2 | Q3 | Q4 |
| --- | --- | --- | --- | --- |
| 2006 | 174 | 40 | 34 | 23 |
| 2007 | 161 | 45 | 36 | 29 |
| 2008 | 140 | 57 | 36 | 38 |
| 2009 | 121 | 72 | 37 | 41 |
| 2010 | 99 | 80 | 39 | 53 |
| 2011 | 85 | 85 | 45 | 56 |
| 2012 | 65 | 81 | 64 | 61 |
| 2013 | 62 | 76 | 64 | 69 |
| 2014 | 33 | 71 | 82 | 85 |
| 2015 | 29 | 85 | 77 | 80 |
| 2016 | 18 | 80 | 86 | 87 |
| 2017 | 19 | 72 | 93 | 87 |
| 2018 | 8 | 69 | 101 | 93 |
| 2019 | 1 | 56 | 111 | 103 |
| 2020 | 0 | 44 | 116 | 111 |

The data for Fig 2

| year | Average index | | | | | Average growth rate(%) | | | | |
| --- | --- | --- | --- | --- | --- | --- | --- | --- | --- | --- |
|  | The national average | The eastern region | The central region | The western region | The northeastern region | The national average | The eastern region | The central region | The western region | The northeastern region |
| 2006 | 0.8031 | 1.0055 | 0.6784 | 0.7208 | 0.8328 | -- | -- | -- | -- | -- |
| 2007 | 0.840 | 1.0483 | 0.7198 | 0.7518 | 0.8657 | 4.58 | 4.26 | 6.1 | 4.3 | 3.94 |
| 2008 | 0.8808 | 1.0941 | 0.7553 | 0.7844 | 0.9021 | 4.72 | 4.32 | 5.52 | 4.32 | 4.08 |
| 2009 | 0.9198 | 1.1401 | 0.7880 | 0.8240 | 0.95422 | 4.62 | 4.28 | 5.12 | 4.56 | 4.64 |
| 2010 | 0.970 | 1.2110 | 0.8143 | 0.8586 | 1.01469 | 4.83 | 4.76 | 4.67 | 4.47 | 5.06 |
| 2011 | 0.9973 | 1.2425 | 0.8517 | 0.8991 | 1.03588 | 4.43 | 4.32 | 4.66 | 4.52 | 4.46 |
| 2012 | 1.066 | 1.329 | 0.893 | 0.953 | 1.1058 | 4.83 | 4.76 | 4.68 | 4.77 | 4.84 |
| 2013 | 1.0947 | 1.3761 | 0.9336 | 0.9742 | 1.1304 | 4.52 | 4.59 | 4.67 | 4.4 | 4.46 |
| 2014 | 1.1623 | 1.4659 | 0.9702 | 1.0464 | 1.14507 | 4.73 | 4.83 | 4.57 | 4.77 | 4.06 |
| 2015 | 1.1662 | 1.4634 | 0.990 | 1.0531 | 1.13991 | 4.23 | 4.26 | 4.29 | 4.3 | 3.55 |
| 2016 | 1.20246 | 1.51219 | 1.00909 | 1.09012 | 1.16209 | 4.12 | 4.17 | 4.05 | 4.22 | 3.39 |
| 2017 | 1.2172 | 1.5426 | 1.0373 | 1.0970 | 1.16595 | 3.85 | 3.97 | 3.94 | 3.89 | 3.11 |
| 2018 | 1.2609 | 1.58771 | 1.0808 | 1.1341 | 1.21545 | 3.83 | 3.88 | 3.96 | 3.85 | 3.2 |
| 2019 | 1.2986 | 1.6210 | 1.116 | 1.1882 | 1.21751 | 3.77 | 3.74 | 3.9 | 3.92 | 2.96 |
| 2020 | 1.3124 | 1.6223 | 1.1307 | 1.211 | 1.20391 | 3.57 | 3.48 | 3.72 | 3.78 | 2.67 |

The data for Fig 3(a)

| year | Overall | Economic  dimension | Social  dimension | Environmental  dimension |
| --- | --- | --- | --- | --- |
| 2006 | 0.80313 | 0.4594 | 0.62999 | 4.05397 |
| 2007 | 0.83995 | 0.49173 | 0.65994 | 4.19034 |
| 2008 | 0.88075 | 0.51976 | 0.69102 | 4.39311 |
| 2009 | 0.91975 | 0.55924 | 0.7215 | 4.53674 |
| 2010 | 0.96996 | 0.59877 | 0.7638 | 4.71971 |
| 2011 | 0.99733 | 0.63734 | 0.78932 | 4.73484 |
| 2012 | 1.0656 | 0.67456 | 0.86213 | 4.84387 |
| 2013 | 1.09466 | 0.73857 | 0.88169 | 4.88159 |
| 2014 | 1.16232 | 0.75874 | 0.95956 | 4.97093 |
| 2015 | 1.16624 | 0.80022 | 0.94801 | 5.04999 |
| 2016 | 1.20246 | 0.832 | 0.98128 | 5.13712 |
| 2017 | 1.21719 | 0.86259 | 0.98981 | 5.18019 |
| 2018 | 1.26087 | 0.89794 | 1.03114 | 5.27952 |
| 2019 | 1.29857 | 0.9268 | 1.06756 | 5.36139 |
| 2020 | 1.31241 | 0.92523 | 1.08008 | 5.44037 |

The data for Fig 3(b)

| year | Eastern region | Central region | Western region | Northeastern region |
| --- | --- | --- | --- | --- |
| 2006 | 0.60909 | 0.38531 | 0.38384 | 0.43587 |
| 2007 | 0.67087 | 0.40749 | 0.39808 | 0.46009 |
| 2008 | 0.71571 | 0.42553 | 0.41808 | 0.48861 |
| 2009 | 0.77439 | 0.44844 | 0.45913 | 0.516 |
| 2010 | 0.83774 | 0.46914 | 0.48826 | 0.56547 |
| 2011 | 0.89956 | 0.49435 | 0.51448 | 0.60647 |
| 2012 | 0.95127 | 0.52755 | 0.54413 | 0.63411 |
| 2013 | 1.07598 | 0.56985 | 0.57365 | 0.67688 |
| 2014 | 1.07786 | 0.60901 | 0.60467 | 0.67131 |
| 2015 | 1.16096 | 0.64842 | 0.63331 | 0.64044 |
| 2016 | 1.21357 | 0.6766 | 0.65554 | 0.64985 |
| 2017 | 1.25735 | 0.70714 | 0.66987 | 0.68499 |
| 2018 | 1.31274 | 0.73499 | 0.70088 | 0.69742 |
| 2019 | 1.33749 | 0.78394 | 0.72785 | 0.69118 |
| 2020 | 1.32082 | 0.79794 | 0.72268 | 0.69846 |

The data for Fig 3(c)

| year | The eastern region | The central region | The western region | The northeast region |
| --- | --- | --- | --- | --- |
| 2006 | 0.82965 | 0.47155 | 0.55658 | 0.68254 |
| 2007 | 0.86733 | 0.4999 | 0.58018 | 0.71153 |
| 2008 | 0.90187 | 0.53735 | 0.60344 | 0.73626 |
| 2009 | 0.93996 | 0.55449 | 0.63296 | 0.78271 |
| 2010 | 1.00409 | 0.58552 | 0.66114 | 0.8301 |
| 2011 | 1.03087 | 0.60213 | 0.69826 | 0.84703 |
| 2012 | 1.12705 | 0.6649 | 0.75427 | 0.92424 |
| 2013 | 1.15917 | 0.68067 | 0.76535 | 0.94082 |
| 2014 | 1.27119 | 0.74307 | 0.84304 | 0.96927 |
| 2015 | 1.24081 | 0.74354 | 0.83601 | 0.96573 |
| 2016 | 1.28602 | 0.76505 | 0.87551 | 0.9826 |
| 2017 | 1.3087 | 0.76665 | 0.88015 | 0.98105 |
| 2018 | 1.34832 | 0.81035 | 0.91538 | 1.03538 |
| 2019 | 1.38149 | 0.8472 | 0.96948 | 1.03715 |
| 2020 | 1.38001 | 0.87377 | 0.99815 | 1.01249 |

The data for Fig 3(d)

| year | The eastern region | The central region | The western region | The northeast region |
| --- | --- | --- | --- | --- |
| 2006 | 4.4547 | 3.87699 | 3.83992 | 3.96534 |
| 2007 | 4.50215 | 4.07974 | 4.01555 | 4.07319 |
| 2008 | 4.69248 | 4.3059 | 4.20521 | 4.28094 |
| 2009 | 4.79614 | 4.45226 | 4.36546 | 4.48192 |
| 2010 | 4.97637 | 4.6561 | 4.49733 | 4.7412 |
| 2011 | 4.97046 | 4.58767 | 4.62489 | 4.75278 |
| 2012 | 5.05099 | 4.72325 | 4.73595 | 4.86449 |
| 2013 | 5.03597 | 4.7308 | 4.8507 | 4.93153 |
| 2014 | 5.12473 | 4.85235 | 4.98373 | 4.83807 |
| 2015 | 5.20019 | 4.9162 | 5.09252 | 4.8939 |
| 2016 | 5.28269 | 5.02462 | 5.14488 | 5.02239 |
| 2017 | 5.3687 | 5.07948 | 5.15792 | 4.99588 |
| 2018 | 5.45003 | 5.21389 | 5.23714 | 5.10199 |
| 2019 | 5.51202 | 5.28164 | 5.37655 | 5.13405 |
| 2020 | 5.60451 | 5.39395 | 5.41426 | 5.19274 |

The data for Fig 4(a)

| id | City name | 2006 | 2007 | 2008 | 2009 | 2010 | 2011 | 2012 | 2013 | 2014 | 2015 | 2016 | 2017 | 2018 | 2019 | 2020 |
| --- | --- | --- | --- | --- | --- | --- | --- | --- | --- | --- | --- | --- | --- | --- | --- | --- |
| 1 | *** | 2.284 | 2.423 | 2.491 | 2.709 | 2.860 | 3.005 | 3.222 | 3.398 | 3.507 | 3.710 | 3.800 | 3.966 | 4.096 | 4.219 | 4.067 |
| 2 | *** | 1.445 | 1.471 | 1.612 | 1.659 | 1.829 | 1.888 | 2.105 | 2.169 | 2.330 | 2.363 | 2.398 | 2.334 | 2.266 | 2.264 | 2.143 |
| 3 | *** | 0.771 | 0.826 | 0.869 | 0.940 | 0.988 | 1.025 | 1.033 | 1.072 | 1.144 | 1.181 | 1.232 | 1.305 | 1.366 | 1.323 | 1.380 |
| 4 | *** | 0.903 | 0.945 | 0.994 | 1.021 | 1.103 | 1.135 | 1.154 | 1.222 | 1.238 | 1.276 | 1.302 | 1.316 | 1.379 | 1.403 | 1.442 |
| 5 | *** | 0.909 | 0.973 | 0.995 | 1.020 | 1.080 | 1.107 | 1.101 | 1.136 | 1.191 | 1.228 | 1.393 | 1.668 | 1.995 | 2.235 | 2.424 |
| 6 | *** | 0.657 | 0.680 | 0.740 | 0.781 | 0.838 | 0.854 | 0.859 | 0.859 | 0.905 | 0.928 | 0.972 | 0.969 | 1.005 | 1.003 | 1.035 |
| 7 | *** | 0.560 | 0.610 | 0.661 | 0.727 | 0.741 | 0.742 | 0.726 | 0.729 | 0.794 | 0.831 | 0.852 | 0.872 | 0.931 | 1.000 | 1.046 |
| 8 | *** | 0.590 | 0.603 | 0.658 | 0.686 | 0.734 | 0.754 | 0.748 | 0.781 | 0.815 | 0.888 | 0.925 | 0.928 | 1.000 | 1.013 | 1.100 |
| 9 | *** | 0.663 | 0.704 | 0.747 | 0.818 | 0.829 | 0.853 | 0.843 | 0.922 | 0.956 | 0.991 | 1.015 | 1.046 | 1.072 | 1.087 | 1.119 |
| 10 | *** | 0.687 | 0.742 | 0.799 | 0.913 | 0.920 | 0.922 | 0.930 | 0.964 | 0.949 | 0.969 | 1.004 | 1.033 | 1.055 | 1.070 | 1.105 |
| 11 | *** | 0.620 | 0.647 | 0.708 | 0.757 | 0.808 | 0.819 | 0.782 | 0.873 | 0.920 | 0.966 | 0.984 | 1.000 | 1.050 | 1.060 | 1.086 |
| 12 | *** | 0.723 | 0.795 | 0.836 | 0.889 | 0.948 | 0.975 | 0.945 | 1.034 | 1.051 | 1.040 | 1.168 | 1.137 | 1.195 | 1.310 | 1.264 |
| 13 | *** | 0.561 | 0.593 | 0.634 | 0.687 | 0.731 | 0.710 | 0.714 | 0.766 | 0.822 | 0.829 | 0.865 | 0.901 | 0.938 | 0.954 | 0.983 |
| 14 | *** | 2.197 | 2.313 | 2.413 | 2.480 | 2.642 | 2.719 | 2.961 | 3.082 | 3.181 | 3.376 | 3.663 | 3.733 | 3.864 | 3.958 | 3.844 |
| 15 | *** | 1.431 | 1.459 | 1.542 | 1.560 | 1.631 | 1.682 | 1.825 | 1.931 | 2.014 | 2.114 | 2.198 | 2.251 | 2.365 | 2.480 | 2.480 |
| 16 | *** | 1.445 | 1.494 | 1.572 | 1.618 | 1.684 | 1.747 | 1.877 | 1.931 | 1.957 | 2.004 | 2.074 | 2.111 | 2.174 | 2.223 | 2.258 |
| 17 | *** | 0.695 | 0.757 | 0.773 | 0.805 | 0.859 | 0.918 | 0.972 | 0.993 | 1.082 | 1.090 | 1.142 | 1.132 | 1.171 | 1.202 | 1.219 |
| 18 | *** | 1.140 | 1.213 | 1.270 | 1.331 | 1.386 | 1.440 | 1.562 | 1.600 | 1.664 | 1.719 | 1.775 | 1.817 | 1.880 | 1.913 | 1.929 |
| 19 | *** | 1.623 | 1.732 | 1.862 | 1.896 | 2.030 | 2.058 | 2.329 | 2.338 | 2.423 | 2.474 | 2.630 | 2.698 | 2.765 | 2.892 | 2.809 |
| 20 | *** | 0.797 | 0.864 | 0.903 | 0.947 | 1.026 | 1.087 | 1.183 | 1.220 | 1.283 | 1.322 | 1.374 | 1.387 | 1.442 | 1.496 | 1.520 |
| 21 | *** | 0.690 | 0.718 | 0.744 | 0.759 | 0.798 | 0.818 | 0.877 | 0.907 | 0.974 | 1.008 | 1.038 | 1.056 | 1.090 | 1.121 | 1.139 |
| 22 | *** | 0.697 | 0.733 | 0.748 | 0.760 | 0.804 | 0.842 | 0.941 | 0.980 | 1.046 | 1.064 | 1.085 | 1.085 | 1.125 | 1.176 | 1.194 |
| 23 | *** | 0.681 | 0.724 | 0.758 | 0.768 | 0.808 | 0.850 | 0.951 | 0.945 | 1.032 | 1.076 | 1.094 | 1.084 | 1.126 | 1.165 | 1.205 |
| 24 | *** | 0.833 | 0.867 | 0.912 | 0.942 | 1.006 | 1.055 | 1.138 | 1.143 | 1.200 | 1.241 | 1.287 | 1.291 | 1.363 | 1.402 | 1.435 |
| 25 | *** | 0.969 | 1.002 | 1.056 | 1.097 | 1.149 | 1.203 | 1.335 | 1.370 | 1.458 | 1.469 | 1.520 | 1.534 | 1.590 | 1.576 | 1.589 |
| 26 | *** | 0.787 | 0.815 | 0.859 | 0.876 | 0.924 | 0.940 | 1.032 | 1.113 | 1.194 | 1.135 | 1.193 | 1.207 | 1.266 | 1.310 | 1.348 |
| 27 | *** | 0.607 | 0.627 | 0.681 | 0.687 | 0.722 | 0.735 | 0.807 | 0.813 | 0.940 | 0.903 | 0.922 | 0.937 | 0.963 | 1.048 | 1.084 |
| 28 | *** | 1.543 | 1.585 | 1.645 | 1.687 | 1.847 | 1.857 | 2.047 | 2.086 | 2.254 | 2.314 | 2.432 | 2.556 | 2.679 | 2.761 | 2.740 |
| 29 | *** | 1.356 | 1.449 | 1.512 | 1.537 | 1.634 | 1.681 | 1.823 | 1.887 | 1.970 | 2.000 | 2.074 | 2.161 | 2.230 | 2.324 | 2.333 |
| 30 | *** | 0.876 | 0.915 | 0.947 | 0.993 | 1.053 | 1.081 | 1.171 | 1.213 | 1.346 | 1.338 | 1.362 | 1.408 | 1.469 | 1.515 | 1.586 |
| 31 | *** | 1.226 | 1.208 | 1.256 | 1.297 | 1.368 | 1.386 | 1.515 | 1.556 | 1.673 | 1.652 | 1.699 | 1.766 | 1.884 | 1.951 | 1.950 |
| 32 | *** | 0.921 | 1.008 | 1.047 | 1.102 | 1.160 | 1.192 | 1.294 | 1.338 | 1.480 | 1.475 | 1.540 | 1.616 | 1.698 | 1.787 | 1.798 |
| 33 | *** | 1.012 | 1.074 | 1.103 | 1.149 | 1.193 | 1.214 | 1.300 | 1.402 | 1.608 | 1.542 | 1.596 | 1.629 | 1.701 | 1.781 | 1.803 |
| 34 | *** | 0.939 | 0.995 | 1.022 | 1.056 | 1.101 | 1.128 | 1.182 | 1.264 | 1.422 | 1.399 | 1.445 | 1.492 | 1.566 | 1.653 | 1.656 |
| 35 | *** | 0.865 | 0.899 | 0.946 | 0.967 | 1.036 | 1.004 | 1.096 | 1.085 | 1.321 | 1.210 | 1.252 | 1.280 | 1.347 | 1.419 | 1.450 |
| 36 | *** | 1.093 | 1.182 | 1.201 | 1.286 | 1.324 | 1.349 | 1.449 | 1.491 | 1.652 | 1.629 | 1.755 | 1.762 | 1.801 | 1.889 | 1.842 |
| 37 | *** | 0.843 | 0.897 | 0.961 | 1.005 | 1.057 | 1.078 | 1.158 | 1.185 | 1.248 | 1.302 | 1.362 | 1.386 | 1.459 | 1.506 | 1.500 |
| 38 | *** | 0.758 | 0.804 | 0.848 | 0.861 | 0.982 | 0.940 | 1.073 | 0.998 | 1.149 | 1.109 | 1.161 | 1.193 | 1.226 | 1.276 | 1.320 |
| 39 | *** | 0.956 | 0.965 | 1.053 | 1.082 | 1.211 | 1.205 | 1.324 | 1.376 | 1.425 | 1.460 | 1.512 | 1.564 | 1.631 | 1.647 | 1.646 |
| 40 | *** | 1.898 | 1.972 | 2.017 | 2.101 | 2.155 | 2.283 | 2.382 | 2.401 | 2.473 | 2.442 | 2.474 | 2.579 | 2.654 | 2.748 | 2.582 |
| 41 | *** | 0.685 | 0.719 | 0.758 | 0.830 | 0.853 | 0.878 | 0.953 | 0.940 | 1.018 | 0.992 | 1.033 | 1.039 | 1.064 | 1.176 | 1.179 |
| 42 | *** | 0.705 | 0.747 | 0.767 | 0.811 | 0.883 | 0.924 | 0.974 | 0.992 | 1.030 | 1.041 | 1.067 | 1.070 | 1.100 | 1.150 | 1.176 |
| 43 | *** | 0.803 | 0.846 | 0.865 | 0.928 | 0.990 | 1.019 | 1.121 | 1.140 | 1.174 | 1.211 | 1.253 | 1.301 | 1.341 | 1.442 | 1.459 |
| 44 | *** | 0.674 | 0.709 | 0.707 | 0.745 | 0.805 | 0.825 | 0.880 | 0.927 | 0.965 | 0.997 | 1.036 | 1.060 | 1.113 | 1.184 | 1.199 |
| 45 | *** | 0.699 | 0.712 | 0.711 | 0.754 | 0.790 | 0.878 | 0.887 | 0.924 | 0.940 | 0.974 | 0.996 | 1.001 | 1.031 | 1.048 | 1.083 |
| 46 | *** | 0.717 | 0.734 | 0.761 | 0.831 | 0.861 | 0.904 | 0.957 | 0.971 | 1.012 | 1.076 | 1.104 | 1.137 | 1.184 | 1.242 | 1.279 |
| 47 | *** | 0.571 | 0.621 | 0.644 | 0.687 | 0.762 | 0.786 | 0.837 | 0.846 | 0.924 | 0.916 | 0.938 | 0.970 | 0.987 | 1.053 | 1.099 |
| 48 | *** | 1.080 | 1.121 | 1.192 | 1.274 | 1.345 | 1.403 | 1.494 | 1.578 | 1.690 | 1.752 | 1.834 | 1.861 | 1.922 | 1.924 | 1.798 |
| 49 | *** | 1.096 | 1.141 | 1.218 | 1.262 | 1.346 | 1.420 | 1.546 | 1.642 | 1.705 | 1.787 | 1.850 | 1.945 | 1.992 | 2.045 | 2.057 |
| 50 | *** | 1.121 | 1.151 | 1.191 | 1.213 | 1.272 | 1.326 | 1.408 | 1.502 | 1.604 | 1.521 | 1.566 | 1.597 | 1.612 | 1.626 | 1.583 |
| 51 | *** | 0.740 | 0.758 | 0.804 | 0.832 | 0.860 | 0.904 | 0.956 | 0.969 | 1.052 | 1.029 | 1.061 | 1.079 | 1.121 | 1.098 | 1.132 |
| 52 | *** | 1.140 | 1.213 | 1.226 | 1.252 | 1.333 | 1.377 | 1.507 | 1.569 | 1.726 | 1.662 | 1.730 | 1.751 | 1.791 | 1.780 | 1.752 |
| 53 | *** | 0.989 | 1.035 | 1.083 | 1.138 | 1.209 | 1.252 | 1.348 | 1.412 | 1.531 | 1.444 | 1.510 | 1.532 | 1.557 | 1.566 | 1.601 |
| 54 | *** | 0.728 | 0.752 | 0.801 | 0.855 | 0.957 | 1.012 | 1.107 | 1.176 | 1.224 | 1.257 | 1.316 | 1.350 | 1.412 | 1.464 | 1.531 |
| 55 | *** | 0.727 | 0.782 | 0.812 | 0.851 | 0.880 | 0.893 | 1.046 | 1.051 | 1.115 | 1.069 | 1.088 | 1.127 | 1.168 | 1.181 | 1.202 |
| 56 | *** | 0.782 | 0.791 | 0.834 | 0.888 | 0.946 | 0.957 | 1.005 | 1.037 | 1.197 | 1.090 | 1.123 | 1.146 | 1.181 | 1.188 | 1.167 |
| 57 | *** | 1.104 | 1.180 | 1.226 | 1.270 | 1.342 | 1.328 | 1.383 | 1.479 | 1.719 | 1.626 | 1.678 | 1.738 | 1.763 | 1.729 | 1.724 |
| 58 | *** | 0.742 | 0.768 | 0.799 | 0.822 | 0.896 | 0.951 | 1.008 | 1.017 | 1.107 | 1.104 | 1.111 | 1.135 | 1.186 | 1.206 | 1.226 |
| 59 | *** | 0.717 | 0.772 | 0.809 | 0.845 | 0.862 | 0.883 | 0.927 | 1.047 | 1.097 | 0.989 | 1.020 | 1.009 | 1.052 | 1.081 | 1.116 |
| 60 | *** | 0.627 | 0.670 | 0.717 | 0.745 | 0.782 | 0.830 | 0.904 | 0.979 | 1.212 | 0.979 | 1.005 | 1.005 | 1.034 | 1.040 | 1.070 |
| 61 | *** | 0.667 | 0.705 | 0.769 | 0.809 | 0.824 | 0.841 | 0.903 | 0.942 | 1.081 | 0.934 | 0.966 | 0.971 | 1.002 | 1.016 | 0.995 |
| 62 | *** | 0.928 | 0.991 | 1.035 | 1.100 | 1.173 | 1.232 | 1.326 | 1.312 | 1.445 | 1.360 | 1.396 | 1.393 | 1.463 | 1.422 | 1.460 |
| 63 | *** | 0.515 | 0.550 | 0.597 | 0.626 | 0.669 | 0.710 | 0.749 | 0.767 | 0.828 | 0.805 | 0.854 | 0.875 | 0.912 | 0.945 | 0.969 |
| 64 | *** | 1.680 | 1.781 | 1.863 | 1.968 | 2.097 | 2.169 | 2.314 | 2.444 | 2.595 | 2.665 | 2.768 | 2.875 | 2.841 | 2.888 | 2.815 |
| 65 | *** | 0.788 | 0.821 | 0.780 | 0.808 | 0.842 | 0.893 | 0.897 | 0.886 | 1.011 | 0.949 | 0.969 | 0.978 | 1.035 | 1.029 | 1.068 |
| 66 | *** | 4.237 | 4.081 | 4.053 | 4.343 | 4.439 | 4.598 | 4.660 | 5.601 | 5.408 | 5.321 | 5.387 | 5.278 | 5.336 | 5.061 | 4.591 |
| 67 | *** | 1.829 | 1.920 | 1.956 | 1.988 | 2.073 | 2.109 | 2.319 | 2.404 | 2.503 | 2.538 | 2.596 | 2.723 | 2.731 | 2.742 | 2.593 |
| 68 | *** | 0.868 | 0.817 | 0.827 | 0.843 | 0.925 | 0.899 | 1.010 | 0.954 | 1.074 | 1.017 | 1.050 | 1.047 | 1.079 | 1.107 | 1.110 |
| 69 | *** | 1.577 | 1.653 | 1.717 | 1.805 | 1.900 | 1.995 | 2.056 | 2.101 | 2.173 | 2.252 | 2.275 | 2.326 | 2.326 | 2.286 | 2.172 |
| 70 | *** | 0.899 | 0.950 | 0.974 | 1.029 | 1.101 | 1.146 | 1.231 | 1.267 | 1.247 | 1.262 | 1.264 | 1.300 | 1.344 | 1.369 | 1.414 |
| 71 | *** | 0.596 | 0.608 | 0.682 | 0.725 | 0.765 | 0.760 | 0.821 | 0.801 | 0.941 | 0.861 | 0.871 | 0.900 | 0.926 | 0.929 | 0.948 |
| 72 | *** | 0.591 | 0.568 | 0.644 | 0.605 | 0.720 | 0.681 | 0.845 | 0.791 | 0.917 | 0.810 | 0.854 | 0.867 | 0.883 | 0.909 | 0.935 |
| 73 | *** | 0.696 | 0.748 | 0.762 | 0.758 | 0.880 | 0.845 | 0.982 | 0.877 | 1.006 | 0.934 | 0.949 | 0.972 | 1.029 | 1.024 | 1.063 |
| 74 | *** | 1.029 | 1.094 | 1.156 | 1.177 | 1.266 | 1.248 | 1.467 | 1.486 | 1.614 | 1.549 | 1.582 | 1.582 | 1.592 | 1.626 | 2.201 |
| 75 | *** | 0.615 | 0.615 | 0.690 | 0.676 | 0.775 | 0.731 | 0.848 | 0.766 | 0.925 | 0.838 | 0.866 | 0.874 | 0.887 | 0.922 | 0.967 |
| 76 | *** | 0.554 | 0.609 | 0.613 | 0.645 | 0.803 | 0.684 | 0.716 | 0.711 | 0.765 | 0.836 | 0.912 | 0.852 | 0.910 | 0.891 | 0.942 |
| 77 | *** | 0.652 | 0.639 | 0.766 | 0.759 | 0.889 | 0.760 | 0.933 | 0.857 | 0.971 | 0.930 | 0.967 | 0.962 | 1.010 | 1.011 | 1.057 |
| 78 | *** | 0.641 | 0.628 | 0.687 | 0.795 | 0.810 | 0.888 | 0.959 | 0.927 | 1.086 | 0.934 | 0.946 | 0.962 | 1.013 | 1.019 | 1.035 |
| 79 | *** | 3.159 | 3.362 | 3.412 | 3.428 | 3.665 | 3.739 | 3.887 | 4.113 | 4.234 | 4.437 | 4.397 | 4.361 | 4.105 | 4.004 | 3.627 |
| 80 | *** | 1.913 | 1.968 | 2.050 | 2.094 | 2.254 | 2.307 | 2.381 | 2.400 | 2.475 | 2.405 | 2.451 | 2.522 | 2.513 | 2.514 | 2.320 |
| 81 | *** | 0.636 | 0.693 | 0.713 | 0.732 | 0.753 | 0.811 | 0.853 | 0.887 | 0.977 | 0.891 | 0.901 | 0.897 | 0.922 | 0.932 | 0.937 |
| 82 | *** | 0.564 | 0.589 | 0.593 | 0.631 | 0.643 | 0.735 | 0.769 | 0.617 | 0.752 | 0.712 | 0.746 | 0.770 | 0.784 | 0.800 | 0.823 |
| 83 | *** | 1.123 | 1.118 | 1.155 | 1.192 | 1.274 | 1.314 | 1.392 | 1.534 | 1.509 | 1.635 | 1.659 | 1.709 | 1.647 | 1.720 | 1.724 |

The data for Fig 4(b)

| id | City name | 2006 | 2007 | 2008 | 2009 | 2010 | 2011 | 2012 | 2013 | 2014 | 2015 | 2016 | 2017 | 2018 | 2019 | 2020 |
| --- | --- | --- | --- | --- | --- | --- | --- | --- | --- | --- | --- | --- | --- | --- | --- | --- |
| 1 | *** | 1.265 | 1.275 | 1.332 | 1.361 | 1.408 | 1.481 | 1.587 | 1.610 | 1.690 | 1.749 | 1.789 | 1.784 | 1.829 | 1.848 | 1.847 |
| 2 | *** | 0.854 | 0.987 | 0.943 | 0.995 | 1.007 | 1.031 | 1.099 | 1.086 | 1.132 | 1.181 | 1.215 | 1.180 | 1.475 | 1.269 | 1.267 |
| 3 | *** | 0.880 | 0.897 | 0.957 | 1.176 | 1.222 | 1.233 | 1.291 | 1.138 | 1.202 | 1.169 | 1.200 | 1.196 | 1.235 | 1.252 | 1.263 |
| 4 | *** | 0.676 | 0.698 | 0.794 | 0.865 | 0.871 | 0.867 | 0.942 | 0.935 | 0.997 | 0.997 | 1.021 | 1.012 | 1.073 | 1.115 | 1.152 |
| 5 | *** | 0.773 | 0.860 | 0.904 | 0.925 | 0.973 | 0.977 | 1.048 | 1.067 | 1.139 | 1.154 | 1.124 | 1.111 | 1.144 | 1.187 | 1.246 |
| 6 | *** | 0.738 | 0.837 | 0.848 | 0.901 | 0.904 | 0.908 | 0.998 | 0.991 | 1.024 | 1.076 | 1.073 | 1.026 | 1.107 | 1.155 | 1.151 |
| 7 | *** | 0.687 | 0.728 | 1.276 | 0.891 | 0.886 | 0.885 | 0.925 | 0.951 | 1.005 | 1.046 | 1.077 | 1.044 | 1.099 | 1.117 | 1.154 |
| 8 | *** | 0.602 | 0.684 | 0.771 | 0.830 | 0.863 | 0.877 | 0.910 | 0.945 | 0.954 | 0.921 | 0.926 | 0.926 | 0.957 | 0.986 | 0.994 |
| 9 | *** | 0.642 | 0.690 | 0.730 | 0.788 | 0.835 | 0.832 | 0.922 | 1.009 | 1.066 | 1.099 | 0.952 | 0.922 | 0.960 | 1.038 | 0.993 |
| 10 | *** | 0.626 | 0.680 | 0.723 | 0.732 | 0.767 | 0.816 | 0.862 | 0.879 | 0.930 | 0.941 | 0.966 | 0.961 | 1.007 | 1.044 | 1.079 |
| 11 | *** | 0.604 | 0.682 | 0.711 | 0.781 | 0.813 | 0.813 | 0.905 | 0.882 | 0.878 | 0.883 | 0.945 | 0.831 | 0.890 | 0.932 | 0.944 |
| 12 | *** | 0.998 | 1.031 | 1.088 | 1.118 | 1.237 | 1.107 | 1.218 | 1.211 | 1.305 | 1.346 | 1.388 | 1.450 | 1.516 | 1.653 | 1.696 |
| 13 | *** | 0.847 | 0.925 | 0.969 | 1.028 | 1.091 | 0.934 | 1.010 | 1.046 | 1.138 | 1.135 | 1.201 | 1.223 | 1.265 | 1.328 | 1.424 |
| 14 | *** | 0.653 | 0.664 | 0.708 | 0.723 | 0.774 | 0.790 | 0.858 | 0.864 | 0.926 | 0.947 | 0.987 | 1.004 | 1.052 | 1.124 | 1.181 |
| 15 | *** | 0.791 | 0.774 | 0.837 | 0.841 | 0.919 | 0.918 | 1.014 | 0.992 | 1.059 | 1.057 | 0.931 | 0.933 | 0.972 | 1.004 | 1.038 |
| 16 | *** | 1.080 | 1.100 | 1.148 | 1.150 | 1.217 | 1.038 | 1.074 | 1.124 | 1.140 | 1.204 | 1.204 | 1.236 | 1.276 | 1.346 | 1.398 |
| 17 | *** | 0.714 | 0.773 | 0.795 | 0.796 | 0.831 | 0.867 | 0.892 | 0.901 | 0.981 | 0.977 | 1.009 | 1.009 | 1.052 | 1.123 | 1.169 |
| 18 | *** | 1.092 | 1.126 | 1.171 | 1.189 | 1.213 | 1.244 | 1.341 | 1.397 | 1.598 | 1.494 | 1.108 | 1.112 | 1.164 | 1.146 | 1.270 |
| 19 | *** | 0.563 | 0.594 | 0.638 | 0.626 | 0.664 | 0.678 | 0.755 | 0.765 | 0.800 | 0.799 | 0.872 | 0.880 | 0.931 | 0.987 | 1.032 |
| 20 | *** | 0.700 | 0.759 | 0.804 | 0.808 | 0.871 | 0.878 | 0.939 | 0.961 | 1.000 | 1.018 | 1.068 | 1.069 | 1.121 | 1.168 | 1.225 |
| 21 | *** | 0.521 | 0.539 | 0.590 | 0.632 | 0.696 | 0.752 | 0.834 | 0.841 | 0.895 | 0.910 | 0.943 | 0.973 | 1.002 | 1.106 | 1.155 |
| 22 | *** | 0.425 | 0.447 | 0.482 | 0.512 | 0.540 | 0.574 | 0.636 | 0.638 | 0.692 | 0.720 | 0.764 | 0.770 | 0.828 | 0.884 | 0.929 |
| 23 | *** | 0.394 | 0.469 | 0.518 | 0.536 | 0.578 | 0.577 | 0.618 | 0.671 | 0.718 | 0.735 | 0.780 | 0.794 | 0.841 | 0.880 | 0.944 |
| 24 | *** | 0.489 | 0.517 | 0.543 | 0.566 | 0.646 | 0.632 | 0.693 | 0.699 | 0.733 | 0.784 | 0.828 | 0.870 | 0.892 | 0.939 | 1.163 |
| 25 | *** | 0.443 | 0.497 | 0.572 | 0.589 | 0.630 | 0.638 | 0.691 | 0.701 | 0.750 | 0.781 | 0.801 | 0.780 | 0.825 | 0.888 | 0.955 |
| 26 | *** | 0.624 | 0.676 | 0.725 | 0.750 | 0.785 | 0.821 | 0.874 | 0.910 | 0.912 | 0.980 | 0.973 | 0.977 | 1.021 | 1.086 | 1.148 |
| 27 | *** | 0.604 | 0.647 | 0.677 | 0.722 | 0.789 | 0.820 | 0.839 | 0.922 | 0.962 | 0.988 | 1.033 | 1.059 | 1.103 | 1.155 | 1.206 |
| 28 | *** | 0.864 | 0.877 | 0.919 | 0.944 | 1.000 | 1.042 | 1.126 | 1.167 | 1.244 | 1.277 | 1.314 | 1.383 | 1.434 | 1.487 | 1.518 |
| 29 | *** | 0.683 | 0.706 | 0.775 | 0.842 | 0.850 | 0.881 | 0.930 | 0.961 | 1.018 | 1.030 | 1.115 | 1.064 | 1.172 | 1.165 | 1.196 |
| 30 | *** | 0.713 | 0.752 | 0.798 | 0.819 | 0.881 | 0.904 | 0.966 | 0.962 | 1.001 | 1.018 | 1.036 | 1.018 | 1.126 | 1.129 | 1.144 |
| 31 | *** | 0.585 | 0.641 | 0.699 | 0.722 | 0.819 | 0.816 | 0.884 | 0.902 | 0.956 | 0.955 | 1.003 | 0.986 | 1.018 | 1.094 | 1.114 |
| 32 | *** | 0.836 | 0.888 | 0.949 | 0.975 | 1.069 | 1.102 | 1.151 | 1.147 | 1.186 | 1.179 | 1.185 | 1.237 | 1.216 | 1.251 | 1.278 |
| 33 | *** | 0.569 | 0.671 | 0.694 | 0.736 | 0.788 | 0.827 | 0.855 | 0.889 | 0.917 | 0.939 | 0.988 | 1.017 | 1.029 | 1.095 | 1.164 |
| 34 | *** | 0.495 | 0.525 | 0.538 | 0.565 | 0.624 | 0.621 | 0.678 | 0.704 | 0.768 | 0.798 | 0.846 | 0.883 | 0.907 | 0.986 | 1.030 |
| 35 | *** | 0.511 | 0.541 | 0.601 | 0.663 | 0.713 | 0.720 | 0.772 | 0.796 | 0.855 | 0.864 | 0.885 | 0.872 | 0.913 | 0.956 | 0.996 |
| 36 | *** | 0.583 | 0.629 | 0.674 | 0.691 | 0.718 | 0.728 | 0.787 | 0.801 | 0.838 | 0.842 | 0.850 | 0.864 | 0.933 | 0.998 | 1.036 |
| 37 | *** | 0.566 | 0.619 | 0.644 | 0.673 | 0.752 | 0.713 | 0.781 | 0.801 | 0.835 | 0.843 | 0.874 | 0.864 | 0.912 | 0.972 | 1.034 |
| 38 | *** | 0.565 | 0.599 | 0.645 | 0.625 | 0.667 | 0.699 | 0.730 | 0.755 | 0.789 | 0.808 | 0.835 | 0.840 | 0.872 | 0.967 | 1.033 |
| 39 | *** | 0.965 | 1.032 | 1.054 | 1.123 | 1.022 | 1.043 | 1.107 | 1.259 | 1.361 | 1.525 | 1.619 | 1.702 | 1.774 | 1.863 | 1.850 |
| 40 | *** | 0.495 | 0.519 | 0.553 | 0.583 | 0.626 | 0.655 | 0.677 | 0.772 | 0.847 | 0.834 | 0.833 | 0.907 | 0.945 | 0.988 | 1.019 |
| 41 | *** | 0.702 | 0.751 | 0.780 | 0.814 | 0.827 | 0.849 | 0.938 | 0.975 | 1.058 | 1.044 | 1.084 | 1.115 | 1.152 | 1.180 | 1.209 |
| 42 | *** | 0.620 | 0.650 | 0.695 | 0.733 | 0.762 | 0.752 | 0.827 | 0.835 | 0.884 | 0.889 | 0.911 | 0.914 | 0.949 | 0.987 | 1.000 |
| 43 | *** | 0.616 | 0.647 | 0.670 | 0.710 | 0.746 | 0.847 | 0.781 | 0.774 | 0.832 | 0.827 | 0.879 | 0.875 | 0.923 | 0.942 | 0.960 |
| 44 | *** | 0.603 | 0.651 | 0.711 | 0.715 | 0.739 | 0.763 | 0.800 | 0.800 | 0.859 | 0.866 | 0.888 | 0.929 | 0.969 | 1.025 | 1.061 |
| 45 | *** | 0.670 | 0.720 | 0.744 | 0.735 | 0.762 | 0.748 | 0.798 | 0.868 | 0.911 | 0.855 | 0.890 | 0.917 | 0.937 | 0.966 | 0.988 |
| 46 | *** | 0.687 | 0.730 | 0.744 | 0.790 | 0.821 | 0.828 | 0.865 | 0.887 | 0.948 | 0.946 | 0.997 | 1.024 | 1.058 | 1.100 | 1.116 |
| 47 | *** | 0.604 | 0.576 | 0.653 | 0.643 | 0.677 | 0.688 | 0.717 | 0.732 | 0.789 | 0.799 | 0.834 | 0.842 | 0.900 | 0.934 | 0.947 |
| 48 | *** | 0.581 | 0.592 | 0.615 | 0.654 | 0.698 | 0.695 | 0.751 | 0.753 | 0.812 | 0.813 | 0.856 | 0.942 | 0.985 | 1.019 | 0.973 |
| 49 | *** | 0.650 | 0.660 | 0.682 | 0.679 | 0.729 | 0.716 | 0.801 | 0.810 | 0.889 | 0.894 | 0.923 | 0.930 | 0.965 | 1.018 | 1.060 |
| 50 | *** | 0.683 | 0.733 | 0.771 | 0.799 | 0.796 | 0.847 | 0.891 | 0.914 | 0.973 | 0.939 | 1.012 | 1.027 | 1.072 | 1.084 | 1.124 |
| 51 | *** | 0.503 | 0.546 | 0.561 | 0.571 | 0.611 | 0.637 | 0.662 | 0.676 | 0.741 | 0.754 | 0.765 | 0.792 | 0.826 | 0.897 | 0.935 |
| 52 | *** | 0.470 | 0.499 | 0.571 | 0.569 | 0.578 | 0.583 | 0.623 | 0.636 | 0.697 | 0.697 | 0.720 | 0.746 | 0.778 | 0.831 | 0.853 |
| 53 | *** | 0.511 | 0.528 | 0.577 | 0.602 | 0.611 | 0.632 | 0.671 | 0.662 | 0.720 | 0.716 | 0.734 | 0.761 | 0.791 | 0.822 | 0.840 |
| 54 | *** | 0.449 | 0.482 | 0.504 | 0.513 | 0.537 | 0.552 | 0.593 | 0.625 | 0.654 | 0.668 | 0.703 | 0.708 | 0.741 | 0.780 | 0.813 |
| 55 | *** | 0.492 | 0.543 | 0.543 | 0.583 | 0.604 | 0.610 | 0.653 | 0.662 | 0.729 | 0.722 | 0.778 | 0.779 | 0.802 | 0.843 | 0.900 |
| 56 | *** | 1.152 | 1.195 | 1.249 | 1.316 | 1.403 | 1.495 | 1.659 | 1.856 | 1.823 | 1.902 | 1.989 | 2.104 | 2.190 | 2.238 | 2.109 |
| 57 | *** | 0.766 | 0.789 | 0.819 | 0.839 | 0.842 | 0.882 | 0.929 | 0.957 | 1.079 | 1.036 | 1.089 | 1.074 | 1.113 | 1.187 | 1.183 |
| 58 | *** | 0.675 | 0.698 | 0.727 | 0.745 | 0.784 | 0.812 | 0.855 | 0.879 | 0.986 | 0.984 | 1.020 | 1.013 | 1.106 | 1.154 | 1.171 |
| 59 | *** | 0.806 | 0.843 | 0.864 | 0.909 | 0.950 | 0.985 | 1.061 | 1.095 | 1.185 | 1.205 | 1.239 | 1.247 | 1.314 | 1.354 | 1.348 |
| 60 | *** | 0.671 | 0.689 | 0.748 | 0.760 | 0.796 | 0.804 | 0.889 | 0.916 | 0.978 | 1.032 | 1.047 | 1.053 | 1.075 | 1.118 | 1.139 |
| 61 | *** | 0.752 | 0.770 | 0.836 | 0.895 | 0.876 | 0.961 | 1.177 | 1.224 | 1.252 | 1.250 | 1.300 | 1.118 | 1.152 | 1.229 | 1.223 |
| 62 | *** | 0.658 | 0.692 | 0.696 | 0.713 | 0.737 | 0.752 | 0.813 | 0.909 | 1.002 | 0.943 | 0.975 | 0.989 | 1.022 | 1.070 | 1.082 |
| 63 | *** | 0.538 | 0.557 | 0.575 | 0.600 | 0.631 | 0.648 | 0.739 | 0.739 | 0.797 | 0.800 | 0.841 | 0.858 | 0.893 | 0.932 | 0.967 |
| 64 | *** | 0.503 | 0.515 | 0.539 | 0.560 | 0.636 | 0.644 | 0.673 | 0.650 | 0.763 | 0.771 | 0.825 | 0.822 | 0.855 | 0.908 | 0.894 |
| 65 | *** | 0.535 | 0.586 | 0.601 | 0.626 | 0.607 | 0.658 | 0.688 | 0.778 | 0.876 | 0.794 | 0.814 | 0.824 | 0.845 | 0.854 | 0.947 |
| 66 | *** | 0.523 | 0.545 | 0.571 | 0.616 | 0.656 | 0.701 | 0.791 | 0.747 | 0.829 | 0.797 | 0.891 | 0.925 | 0.957 | 1.008 | 0.992 |
| 67 | *** | 0.566 | 0.572 | 0.609 | 0.643 | 0.679 | 0.661 | 0.721 | 0.720 | 0.792 | 0.781 | 0.817 | 0.816 | 0.847 | 0.890 | 0.909 |
| 68 | *** | 0.962 | 1.030 | 1.073 | 1.141 | 1.241 | 1.289 | 1.413 | 1.473 | 1.577 | 1.638 | 1.722 | 1.753 | 1.836 | 1.933 | 1.968 |
| 69 | *** | 0.718 | 0.763 | 0.799 | 0.831 | 0.925 | 0.922 | 1.005 | 0.983 | 1.076 | 1.118 | 1.157 | 1.197 | 1.261 | 1.237 | 1.255 |
| 70 | *** | 0.702 | 0.759 | 0.800 | 0.788 | 0.849 | 0.860 | 0.932 | 0.951 | 1.065 | 1.082 | 1.128 | 1.165 | 1.185 | 1.206 | 1.196 |
| 71 | *** | 0.528 | 0.579 | 0.608 | 0.632 | 0.704 | 0.733 | 0.755 | 0.768 | 0.852 | 0.873 | 0.928 | 0.905 | 0.927 | 0.974 | 1.003 |
| 72 | *** | 0.460 | 0.501 | 0.559 | 0.584 | 0.626 | 0.656 | 0.657 | 0.671 | 0.687 | 0.731 | 0.806 | 0.784 | 0.816 | 0.845 | 0.871 |
| 73 | *** | 0.689 | 0.674 | 0.747 | 0.796 | 0.828 | 0.809 | 0.845 | 0.789 | 0.984 | 0.947 | 0.998 | 1.045 | 1.036 | 1.054 | 1.079 |
| 74 | *** | 0.567 | 0.606 | 0.640 | 0.714 | 0.760 | 0.786 | 0.874 | 0.919 | 0.893 | 0.935 | 0.949 | 0.967 | 1.036 | 1.076 | 1.113 |
| 75 | *** | 0.649 | 0.647 | 0.699 | 0.705 | 0.718 | 0.744 | 0.801 | 0.793 | 0.849 | 0.895 | 0.931 | 0.923 | 0.934 | 0.946 | 0.958 |
| 76 | *** | 0.569 | 0.557 | 0.596 | 0.612 | 0.661 | 0.660 | 0.749 | 0.715 | 0.804 | 0.823 | 0.857 | 0.879 | 0.911 | 0.935 | 0.967 |
| 77 | *** | 0.603 | 0.622 | 0.643 | 0.693 | 0.714 | 0.763 | 0.805 | 0.853 | 0.929 | 0.932 | 0.952 | 0.963 | 0.953 | 1.020 | 0.995 |
| 78 | *** | 0.499 | 0.536 | 0.571 | 0.586 | 0.620 | 0.616 | 0.694 | 0.706 | 0.783 | 0.816 | 0.846 | 0.861 | 0.884 | 0.914 | 0.933 |
| 79 | *** | 0.547 | 0.591 | 0.601 | 0.608 | 0.661 | 0.677 | 0.767 | 0.750 | 0.800 | 0.779 | 0.833 | 0.883 | 0.944 | 0.911 | 0.936 |
| 80 | *** | 0.565 | 0.595 | 0.597 | 0.652 | 0.661 | 0.670 | 0.720 | 0.720 | 0.745 | 0.776 | 0.829 | 0.898 | 0.999 | 0.974 | 1.002 |

The data for Fig 4(c)

| id | City name | 2006 | 2007 | 2008 | 2009 | 2010 | 2011 | 2012 | 2013 | 2014 | 2015 | 2016 | 2017 | 2018 | 2019 | 2020 |
| --- | --- | --- | --- | --- | --- | --- | --- | --- | --- | --- | --- | --- | --- | --- | --- | --- |
| 1 | *** | 1.144 | 1.189 | 1.279 | 1.389 | 1.413 | 1.444 | 1.438 | 1.520 | 1.546 | 1.657 | 1.714 | 1.748 | 1.786 | 1.776 | 1.755 |
| 2 | *** | 1.301 | 1.364 | 1.429 | 1.481 | 1.567 | 1.600 | 1.691 | 1.718 | 1.818 | 1.834 | 1.896 | 1.935 | 2.023 | 2.048 | 1.995 |
| 3 | *** | 1.728 | 1.510 | 1.640 | 1.660 | 1.601 | 1.707 | 1.744 | 1.767 | 1.923 | 1.911 | 2.044 | 2.052 | 2.122 | 2.241 | 2.232 |
| 4 | *** | 0.679 | 0.694 | 0.736 | 0.756 | 0.794 | 0.820 | 0.898 | 0.931 | 0.936 | 1.012 | 1.037 | 1.027 | 1.095 | 1.116 | 1.132 |
| 5 | *** | 0.758 | 0.807 | 0.857 | 0.892 | 0.940 | 0.976 | 1.031 | 1.051 | 1.128 | 1.072 | 1.161 | 1.156 | 1.178 | 1.311 | 1.197 |
| 6 | *** | 1.018 | 1.182 | 1.301 | 1.413 | 1.599 | 1.663 | 1.864 | 1.963 | 2.056 | 2.098 | 2.120 | 2.143 | 2.195 | 2.284 | 2.306 |
| 7 | *** | 0.873 | 0.878 | 0.898 | 0.926 | 0.959 | 1.042 | 1.167 | 1.189 | 1.225 | 1.212 | 1.225 | 1.288 | 1.211 | 1.199 | 1.214 |
| 8 | *** | 0.737 | 0.809 | 0.843 | 0.865 | 0.899 | 0.915 | 0.957 | 1.026 | 1.099 | 1.100 | 1.131 | 1.100 | 1.080 | 1.144 | 1.184 |
| 9 | *** | 0.754 | 0.808 | 0.848 | 0.849 | 0.899 | 0.913 | 1.001 | 1.003 | 1.053 | 1.096 | 1.152 | 1.151 | 1.195 | 1.194 | 1.277 |
| 10 | *** | 0.728 | 0.762 | 0.831 | 0.891 | 0.925 | 0.966 | 1.004 | 1.030 | 1.100 | 1.115 | 1.162 | 1.179 | 1.235 | 1.297 | 1.303 |
| 11 | *** | 0.754 | 0.779 | 0.824 | 0.885 | 0.922 | 0.916 | 1.015 | 1.008 | 1.075 | 1.115 | 1.147 | 1.165 | 1.217 | 1.259 | 1.268 |
| 12 | *** | 0.650 | 0.671 | 0.705 | 0.713 | 0.752 | 0.773 | 0.814 | 0.836 | 0.874 | 0.904 | 0.937 | 0.965 | 1.005 | 1.024 | 1.046 |
| 13 | *** | 0.537 | 0.558 | 0.572 | 0.625 | 0.680 | 0.730 | 0.732 | 0.742 | 0.787 | 0.813 | 0.841 | 0.797 | 0.859 | 0.897 | 0.911 |
| 14 | *** | 0.640 | 0.627 | 0.668 | 0.732 | 0.773 | 0.980 | 0.876 | 0.885 | 0.919 | 0.951 | 0.970 | 0.980 | 1.046 | 1.088 | 1.134 |
| 15 | *** | 0.664 | 0.670 | 0.701 | 0.736 | 0.836 | 0.832 | 0.928 | 0.934 | 1.056 | 1.015 | 1.041 | 1.043 | 1.087 | 1.157 | 1.174 |
| 16 | *** | 0.440 | 0.526 | 0.518 | 0.597 | 0.638 | 0.920 | 0.718 | 0.757 | 0.759 | 0.813 | 0.859 | 0.819 | 0.872 | 0.906 | 0.948 |
| 17 | *** | 0.521 | 0.518 | 0.507 | 0.588 | 0.646 | 0.634 | 0.684 | 0.684 | 0.717 | 0.740 | 0.752 | 0.769 | 0.751 | 0.810 | 0.824 |
| 18 | *** | 0.535 | 0.502 | 0.524 | 0.563 | 0.606 | 0.700 | 0.714 | 0.724 | 0.728 | 0.760 | 0.809 | 0.771 | 0.856 | 0.922 | 0.973 |
| 19 | *** | 0.504 | 0.460 | 0.496 | 0.591 | 0.626 | 0.629 | 0.646 | 0.669 | 0.666 | 0.739 | 0.792 | 0.809 | 0.869 | 0.904 | 0.953 |
| 20 | *** | 0.452 | 0.471 | 0.519 | 0.562 | 0.580 | 0.550 | 0.590 | 0.600 | 0.657 | 0.699 | 0.738 | 0.725 | 0.771 | 0.847 | 0.866 |
| 21 | *** | 0.438 | 0.494 | 0.536 | 0.601 | 0.631 | 0.665 | 0.717 | 0.736 | 0.773 | 0.786 | 0.804 | 0.783 | 0.831 | 0.848 | 0.887 |
| 22 | *** | 0.735 | 0.812 | 0.874 | 0.944 | 1.050 | 1.168 | 1.272 | 1.304 | 1.496 | 1.473 | 1.541 | 1.564 | 1.575 | 1.623 | 1.645 |
| 23 | *** | 1.028 | 1.095 | 1.168 | 1.230 | 1.323 | 1.418 | 1.532 | 1.595 | 1.712 | 1.750 | 1.723 | 1.813 | 1.913 | 1.996 | 1.986 |
| 24 | *** | 0.556 | 0.593 | 0.611 | 0.642 | 0.667 | 0.741 | 0.747 | 0.768 | 0.856 | 0.847 | 0.872 | 0.871 | 0.939 | 1.013 | 1.033 |
| 25 | *** | 1.037 | 1.054 | 1.081 | 1.107 | 1.140 | 1.227 | 1.271 | 1.315 | 1.384 | 1.368 | 1.379 | 1.584 | 1.433 | 1.468 | 1.449 |
| 26 | *** | 0.538 | 0.514 | 0.534 | 0.568 | 0.606 | 0.654 | 0.711 | 0.753 | 0.812 | 0.831 | 0.878 | 0.895 | 0.940 | 1.001 | 1.045 |
| 27 | *** | 0.588 | 0.601 | 0.624 | 0.677 | 0.734 | 0.816 | 0.841 | 0.947 | 0.990 | 0.920 | 0.941 | 0.951 | 0.990 | 1.085 | 1.116 |
| 28 | *** | 0.632 | 0.653 | 0.665 | 0.727 | 0.775 | 0.848 | 0.894 | 0.902 | 0.967 | 0.982 | 1.007 | 1.027 | 1.077 | 1.101 | 1.141 |
| 29 | *** | 0.594 | 0.647 | 0.679 | 0.714 | 0.751 | 0.787 | 0.839 | 0.854 | 0.914 | 0.913 | 0.944 | 0.932 | 0.958 | 0.986 | 1.040 |
| 30 | *** | 0.490 | 0.546 | 0.586 | 0.607 | 0.617 | 0.657 | 0.709 | 0.754 | 0.968 | 0.858 | 0.895 | 0.864 | 0.935 | 0.947 | 1.058 |
| 31 | *** | 0.441 | 0.476 | 0.503 | 0.552 | 0.579 | 0.635 | 0.671 | 0.668 | 0.807 | 0.774 | 0.814 | 0.802 | 0.862 | 0.895 | 0.938 |
| 32 | *** | 0.674 | 0.686 | 0.712 | 0.745 | 0.763 | 0.802 | 0.875 | 0.903 | 0.968 | 0.944 | 0.969 | 0.984 | 1.019 | 1.079 | 1.110 |
| 33 | *** | 0.514 | 0.581 | 0.585 | 0.609 | 0.625 | 0.670 | 0.723 | 0.714 | 0.792 | 0.796 | 0.848 | 0.839 | 0.872 | 0.899 | 0.937 |
| 34 | *** | 0.536 | 0.560 | 0.567 | 0.583 | 0.627 | 0.684 | 0.739 | 0.728 | 0.796 | 1.047 | 0.854 | 0.886 | 0.924 | 0.989 | 0.987 |
| 35 | *** | 0.511 | 0.532 | 0.592 | 0.607 | 0.640 | 0.689 | 0.730 | 0.744 | 0.836 | 0.814 | 0.844 | 0.858 | 0.952 | 0.990 | 1.032 |
| 36 | *** | 0.519 | 0.546 | 0.580 | 0.579 | 0.566 | 0.567 | 0.578 | 0.633 | 0.734 | 0.711 | 0.753 | 0.759 | 0.837 | 0.879 | 0.941 |
| 37 | *** | 0.665 | 0.688 | 0.720 | 0.748 | 0.777 | 0.790 | 0.936 | 0.898 | 0.979 | 1.032 | 1.059 | 1.034 | 1.074 | 1.217 | 1.228 |
| 38 | *** | 0.496 | 0.523 | 0.554 | 0.567 | 0.609 | 0.636 | 0.687 | 0.689 | 0.760 | 0.768 | 0.824 | 0.799 | 0.863 | 0.886 | 0.918 |
| 39 | *** | 0.892 | 0.970 | 0.974 | 1.042 | 1.149 | 1.176 | 1.275 | 1.372 | 1.433 | 1.522 | 1.571 | 1.634 | 1.681 | 1.757 | 1.821 |
| 40 | *** | 0.518 | 0.521 | 0.559 | 0.598 | 0.628 | 0.663 | 0.718 | 0.981 | 1.085 | 0.860 | 0.862 | 0.885 | 0.918 | 0.956 | 0.982 |
| 41 | *** | 0.519 | 0.527 | 0.791 | 0.591 | 0.592 | 0.651 | 0.691 | 0.731 | 0.829 | 0.815 | 0.910 | 0.916 | 0.991 | 1.036 | 1.118 |
| 42 | *** | 0.428 | 0.462 | 0.485 | 0.528 | 0.564 | 0.617 | 0.647 | 0.671 | 0.721 | 0.814 | 0.870 | 0.877 | 0.892 | 0.952 | 0.994 |
| 43 | *** | 1.033 | 1.070 | 1.088 | 1.287 | 1.249 | 1.316 | 1.495 | 1.436 | 1.594 | 1.505 | 1.601 | 1.623 | 1.680 | 1.761 | 1.775 |
| 44 | *** | 0.541 | 0.591 | 0.612 | 0.642 | 0.661 | 0.676 | 0.757 | 0.783 | 0.834 | 0.816 | 0.843 | 0.788 | 0.890 | 0.944 | 0.967 |
| 45 | *** | 0.705 | 0.732 | 0.757 | 0.823 | 0.815 | 0.805 | 0.882 | 0.905 | 0.978 | 1.024 | 1.065 | 1.088 | 1.127 | 1.251 | 1.278 |
| 46 | *** | 0.582 | 0.600 | 0.613 | 0.657 | 0.661 | 0.700 | 0.676 | 0.698 | 0.749 | 0.762 | 0.849 | 0.825 | 0.866 | 0.953 | 0.989 |
| 47 | *** | 0.367 | 0.394 | 0.443 | 0.453 | 0.506 | 0.504 | 0.532 | 0.692 | 0.584 | 0.653 | 0.689 | 0.713 | 0.736 | 0.792 | 0.836 |
| 48 | *** | 0.657 | 0.692 | 0.725 | 0.753 | 0.778 | 0.794 | 0.845 | 0.884 | 0.893 | 0.906 | 0.917 | 0.913 | 0.941 | 1.052 | 1.074 |
| 49 | *** | 0.466 | 0.533 | 0.565 | 0.571 | 0.639 | 0.600 | 0.632 | 0.653 | 0.686 | 0.709 | 0.763 | 0.750 | 0.823 | 0.909 | 0.959 |
| 50 | *** | 0.419 | 0.463 | 0.454 | 0.478 | 0.511 | 0.582 | 0.641 | 0.675 | 0.681 | 0.711 | 0.770 | 0.760 | 0.770 | 0.828 | 0.848 |
| 51 | *** | 1.033 | 1.066 | 1.137 | 1.183 | 1.254 | 1.338 | 1.432 | 1.454 | 1.538 | 1.574 | 1.589 | 1.596 | 1.592 | 1.737 | 1.797 |
| 52 | *** | 0.946 | 0.936 | 0.995 | 1.056 | 1.073 | 1.156 | 1.214 | 1.172 | 1.236 | 1.205 | 1.229 | 1.246 | 1.300 | 1.308 | 1.325 |
| 53 | *** | 0.737 | 0.774 | 0.787 | 0.815 | 0.833 | 0.878 | 0.931 | 0.932 | 0.965 | 1.004 | 1.006 | 1.019 | 1.050 | 1.232 | 1.090 |
| 54 | *** | 0.614 | 0.639 | 0.682 | 0.701 | 0.738 | 0.761 | 0.850 | 0.864 | 0.906 | 0.917 | 0.922 | 0.949 | 0.951 | 1.017 | 1.018 |
| 55 | *** | 0.525 | 0.530 | 0.598 | 0.623 | 0.689 | 0.730 | 0.808 | 0.777 | 0.828 | 0.841 | 0.859 | 0.845 | 0.880 | 0.895 | 0.920 |
| 56 | *** | 0.667 | 0.706 | 0.784 | 0.808 | 0.834 | 0.863 | 0.905 | 0.978 | 1.151 | 0.977 | 0.992 | 0.976 | 1.023 | 1.073 | 1.105 |
| 57 | *** | 0.566 | 0.613 | 0.622 | 0.732 | 0.767 | 0.810 | 0.866 | 0.889 | 0.934 | 0.834 | 0.871 | 0.862 | 0.910 | 0.939 | 0.963 |
| 58 | *** | 0.586 | 0.662 | 0.749 | 0.820 | 0.901 | 0.984 | 1.066 | 1.051 | 1.272 | 1.067 | 1.092 | 1.070 | 1.163 | 1.176 | 1.188 |
| 59 | *** | 0.596 | 0.629 | 0.640 | 0.667 | 0.638 | 0.681 | 0.751 | 0.784 | 0.829 | 0.880 | 0.896 | 0.876 | 0.845 | 0.896 | 0.903 |
| 60 | *** | 0.489 | 0.523 | 0.548 | 0.595 | 0.623 | 0.677 | 0.704 | 0.816 | 0.742 | 0.747 | 0.751 | 0.767 | 0.774 | 0.841 | 0.827 |
| 61 | *** | 1.112 | 1.110 | 1.109 | 1.183 | 1.224 | 1.258 | 1.299 | 1.236 | 1.300 | 1.458 | 1.563 | 1.624 | 1.688 | 1.725 | 1.740 |
| 62 | *** | 0.891 | 0.969 | 0.976 | 0.932 | 1.004 | 0.988 | 1.061 | 1.050 | 1.118 | 1.181 | 1.222 | 1.265 | 1.336 | 1.494 | 1.454 |
| 63 | *** | 0.612 | 0.667 | 0.710 | 0.713 | 0.755 | 0.735 | 0.816 | 0.822 | 0.873 | 0.885 | 0.913 | 0.987 | 0.971 | 0.983 | 1.041 |
| 64 | *** | 0.532 | 0.553 | 0.600 | 0.614 | 0.622 | 0.604 | 0.642 | 0.656 | 0.692 | 0.717 | 0.743 | 0.771 | 0.790 | 0.816 | 0.852 |
| 65 | *** | 0.709 | 0.702 | 0.724 | 0.743 | 0.793 | 0.750 | 0.827 | 0.839 | 1.345 | 0.920 | 0.925 | 0.912 | 0.926 | 0.943 | 1.000 |
| 66 | *** | 1.146 | 1.216 | 0.771 | 0.793 | 0.786 | 0.810 | 0.897 | 0.992 | 1.094 | 1.058 | 1.086 | 1.128 | 1.011 | 1.070 | 1.071 |
| 67 | *** | 0.549 | 0.572 | 0.610 | 0.648 | 0.653 | 0.617 | 0.665 | 0.705 | 0.742 | 0.797 | 0.824 | 0.845 | 0.905 | 0.921 | 0.920 |
| 68 | *** | 0.845 | 0.868 | 0.883 | 0.900 | 0.917 | 0.956 | 1.025 | 0.941 | 0.981 | 1.138 | 1.415 | 1.103 | 1.089 | 1.136 | 1.243 |
| 69 | *** | 0.457 | 0.503 | 0.575 | 0.594 | 0.577 | 0.622 | 0.699 | 0.712 | 0.728 | 0.743 | 0.773 | 0.773 | 0.821 | 0.846 | 0.878 |
| 70 | *** | 0.425 | 0.438 | 0.468 | 0.498 | 0.507 | 0.539 | 0.598 | 0.603 | 0.663 | 0.714 | 0.740 | 0.742 | 0.765 | 0.793 | 0.845 |
| 71 | *** | 1.083 | 1.108 | 1.215 | 1.232 | 1.214 | 1.384 | 1.566 | 1.309 | 1.425 | 1.546 | 1.566 | 1.573 | 1.568 | 1.669 | 1.671 |
| 72 | *** | 1.528 | 1.531 | 1.565 | 1.412 | 1.481 | 1.490 | 1.563 | 1.582 | 1.583 | 1.654 | 1.684 | 1.727 | 1.791 | 1.850 | 1.853 |
| 73 | *** | 1.355 | 1.512 | 1.528 | 1.812 | 1.598 | 1.629 | 1.714 | 1.532 | 1.678 | 1.711 | 1.749 | 1.758 | 1.770 | 1.790 | 1.793 |
| 74 | *** | 0.757 | 0.851 | 0.893 | 0.919 | 1.167 | 1.150 | 1.159 | 1.171 | 1.296 | 1.265 | 1.293 | 1.029 | 1.087 | 1.120 | 1.122 |
| 75 | *** | 0.892 | 0.993 | 0.978 | 1.076 | 1.085 | 0.979 | 0.849 | 0.885 | 0.915 | 0.862 | 0.962 | 0.993 | 0.991 | 1.010 | 1.170 |
| 76 | *** | 1.600 | 1.525 | 1.532 | 1.611 | 1.634 | 1.699 | 1.783 | 1.867 | 1.962 | 1.970 | 2.001 | 2.306 | 2.428 | 2.505 | 2.422 |

The data for Fig 4(d)

| id | City name | 2006 | 2007 | 2008 | 2009 | 2010 | 2011 | 2012 | 2013 | 2014 | 2015 | 2016 | 2017 | 2018 | 2019 | 2020 |
| --- | --- | --- | --- | --- | --- | --- | --- | --- | --- | --- | --- | --- | --- | --- | --- | --- |
| 1 | *** | 1.273 | 1.329 | 1.354 | 1.412 | 1.497 | 1.566 | 1.649 | 1.678 | 1.758 | 1.731 | 1.741 | 1.798 | 2.387 | 1.846 | 1.827 |
| 2 | *** | 1.190 | 1.265 | 1.338 | 1.406 | 1.483 | 1.547 | 1.655 | 1.703 | 1.738 | 1.723 | 1.785 | 1.836 | 1.884 | 1.830 | 1.792 |
| 3 | *** | 0.894 | 0.923 | 0.954 | 0.991 | 1.055 | 1.108 | 1.147 | 1.168 | 1.194 | 1.211 | 1.234 | 1.239 | 1.305 | 1.318 | 1.308 |
| 4 | *** | 1.067 | 1.077 | 1.117 | 1.109 | 1.166 | 1.195 | 1.267 | 1.271 | 1.385 | 1.337 | 1.334 | 1.322 | 1.329 | 1.323 | 1.269 |
| 5 | *** | 1.162 | 1.202 | 1.251 | 1.272 | 1.322 | 1.349 | 1.418 | 1.429 | 1.464 | 1.410 | 1.440 | 1.467 | 1.473 | 1.497 | 1.451 |
| 6 | *** | 0.808 | 0.836 | 0.849 | 0.877 | 1.004 | 0.989 | 1.052 | 1.104 | 1.129 | 1.131 | 1.121 | 1.103 | 1.136 | 1.097 | 1.060 |
| 7 | *** | 0.801 | 0.809 | 0.831 | 0.874 | 0.923 | 1.021 | 0.994 | 1.022 | 1.082 | 1.111 | 1.144 | 1.118 | 1.138 | 1.469 | 1.479 |
| 8 | *** | 0.890 | 0.950 | 0.983 | 1.009 | 1.097 | 1.088 | 1.190 | 1.210 | 1.204 | 1.208 | 1.305 | 1.341 | 1.426 | 1.437 | 1.441 |
| 9 | *** | 0.745 | 0.776 | 0.814 | 0.845 | 0.912 | 0.933 | 0.962 | 0.940 | 1.006 | 1.026 | 1.055 | 1.088 | 1.046 | 1.096 | 1.078 |
| 10 | *** | 0.865 | 0.942 | 0.974 | 1.142 | 1.234 | 1.124 | 1.186 | 1.224 | 1.205 | 1.181 | 1.191 | 1.195 | 1.243 | 1.266 | 1.288 |
| 11 | *** | 1.105 | 1.130 | 1.134 | 1.418 | 1.483 | 1.718 | 1.819 | 1.810 | 1.472 | 1.443 | 1.477 | 1.513 | 1.553 | 1.619 | 1.599 |
| 12 | *** | 0.637 | 0.698 | 0.729 | 0.738 | 0.806 | 0.872 | 0.884 | 0.871 | 0.853 | 0.894 | 0.901 | 0.928 | 0.916 | 0.908 | 0.907 |
| 13 | *** | 0.565 | 0.582 | 0.626 | 0.620 | 0.718 | 0.694 | 0.787 | 0.798 | 0.834 | 0.862 | 0.878 | 0.897 | 0.905 | 0.936 | 0.935 |
| 14 | *** | 0.783 | 0.794 | 0.830 | 0.829 | 0.846 | 0.847 | 0.977 | 1.522 | 0.992 | 0.988 | 0.981 | 0.995 | 1.008 | 1.063 | 1.091 |
| 15 | *** | 0.967 | 0.992 | 1.048 | 1.105 | 1.163 | 1.213 | 1.289 | 1.263 | 1.337 | 1.331 | 1.370 | 1.397 | 1.484 | 1.482 | 1.515 |
| 16 | *** | 0.853 | 0.878 | 0.954 | 0.980 | 1.062 | 1.034 | 1.137 | 1.130 | 1.196 | 1.162 | 1.206 | 1.199 | 1.245 | 1.219 | 1.162 |
| 17 | *** | 0.668 | 0.632 | 0.698 | 0.716 | 0.773 | 0.755 | 0.848 | 0.867 | 0.897 | 0.855 | 0.898 | 0.894 | 0.937 | 0.979 | 0.853 |
| 18 | *** | 0.805 | 0.811 | 0.842 | 0.914 | 0.921 | 0.898 | 0.988 | 0.971 | 1.007 | 1.006 | 1.012 | 1.017 | 1.061 | 1.093 | 1.071 |
| 19 | *** | 0.718 | 0.729 | 0.768 | 0.823 | 0.877 | 0.943 | 1.003 | 0.997 | 1.014 | 1.093 | 1.097 | 1.082 | 1.055 | 1.096 | 1.113 |
| 20 | *** | 0.805 | 0.868 | 0.892 | 0.982 | 1.027 | 1.025 | 1.104 | 1.089 | 1.121 | 1.090 | 1.145 | 1.111 | 1.203 | 1.183 | 1.170 |
| 21 | *** | 0.609 | 0.658 | 0.722 | 0.739 | 0.798 | 0.802 | 0.891 | 0.916 | 0.942 | 0.968 | 0.977 | 0.969 | 0.997 | 0.986 | 0.947 |
| 22 | *** | 0.667 | 0.700 | 0.728 | 0.729 | 0.765 | 0.775 | 0.870 | 0.891 | 0.936 | 0.918 | 0.952 | 0.951 | 0.963 | 1.003 | 0.995 |
| 23 | *** | 0.877 | 0.891 | 0.944 | 0.994 | 1.070 | 1.098 | 1.175 | 1.228 | 1.283 | 1.348 | 1.382 | 1.390 | 1.460 | 1.454 | 1.404 |
| 24 | *** | 0.649 | 0.666 | 0.681 | 0.836 | 0.885 | 0.797 | 0.890 | 0.865 | 0.937 | 0.935 | 1.016 | 0.903 | 0.924 | 0.875 | 0.921 |
| 25 | *** | 0.755 | 0.774 | 0.799 | 0.886 | 0.894 | 0.991 | 1.025 | 0.943 | 0.983 | 0.946 | 0.996 | 0.986 | 1.005 | 1.020 | 1.036 |
| 26 | *** | 0.786 | 0.823 | 0.853 | 0.894 | 0.937 | 0.931 | 0.980 | 0.983 | 1.007 | 1.025 | 1.102 | 1.103 | 1.139 | 1.192 | 1.179 |
| 27 | *** | 1.057 | 1.141 | 1.233 | 1.227 | 1.378 | 1.397 | 1.448 | 1.427 | 1.609 | 1.578 | 1.423 | 1.406 | 1.469 | 1.427 | 1.369 |
| 28 | *** | 0.795 | 0.817 | 0.841 | 0.909 | 0.967 | 1.000 | 1.091 | 1.060 | 1.130 | 1.098 | 1.178 | 1.145 | 1.171 | 1.167 | 1.135 |
| 29 | *** | 0.745 | 0.800 | 0.813 | 0.862 | 0.894 | 0.838 | 0.907 | 0.972 | 1.011 | 0.945 | 0.947 | 0.966 | 1.011 | 0.997 | 1.029 |
| 30 | *** | 0.758 | 0.799 | 0.784 | 0.824 | 0.851 | 0.860 | 0.951 | 0.985 | 0.991 | 0.944 | 1.015 | 1.047 | 1.074 | 1.080 | 1.092 |
| 31 | *** | 0.758 | 0.773 | 0.836 | 0.856 | 0.889 | 0.938 | 0.956 | 0.975 | 1.002 | 1.035 | 1.097 | 1.093 | 1.119 | 1.123 | 1.102 |
| 32 | *** | 0.594 | 0.638 | 0.646 | 0.717 | 0.772 | 0.801 | 0.850 | 0.861 | 0.923 | 0.941 | 0.788 | 0.812 | 0.829 | 0.880 | 0.906 |

The data for Fig 5(a)

| year | 2006 | 2007 | 2008 | 2009 | 2010 | 2011 | 2012 | 2013 | 2014 | 2015 | 2016 | 2017 | 2018 | 2019 | 2020 |
| --- | --- | --- | --- | --- | --- | --- | --- | --- | --- | --- | --- | --- | --- | --- | --- |
| Overall differences | 0.2146 | 0.2104 | 0.2046 | 0.2034 | 0.2016 | 0.202 | 0.2019 | 0.2054 | 0.1992 | 0.1998 | 0.1975 | 0.2018 | 0.1986 | 0.1924 | 0.182 |

The data for Fig 5(b)

| year | The eastern region | The central region | The western region | The northeast region |
| --- | --- | --- | --- | --- |
| 2006 | 0.2524 | 0.1159 | 0.2045 | 0.1352 |
| 2007 | 0.2497 | 0.1173 | 0.198 | 0.1319 |
| 2008 | 0.2431 | 0.1157 | 0.1907 | 0.1327 |
| 2009 | 0.2413 | 0.1184 | 0.1881 | 0.1295 |
| 2010 | 0.2375 | 0.1177 | 0.1834 | 0.1249 |
| 2011 | 0.241 | 0.127 | 0.1823 | 0.121 |
| 2012 | 0.2411 | 0.1196 | 0.1821 | 0.1221 |
| 2013 | 0.2537 | 0.1247 | 0.1727 | 0.1243 |
| 2014 | 0.2398 | 0.1135 | 0.1762 | 0.1198 |
| 2015 | 0.2495 | 0.1107 | 0.1715 | 0.1218 |
| 2016 | 0.2482 | 0.1101 | 0.1684 | 0.1138 |
| 2017 | 0.2501 | 0.1136 | 0.1751 | 0.1132 |
| 2018 | 0.2443 | 0.1293 | 0.1688 | 0.1141 |
| 2019 | 0.2413 | 0.1155 | 0.1658 | 0.109 |
| 2020 | 0.2268 | 0.1567 | 0.1144 | 0.1043 |

The data for Fig 5(c)

| year | Eastern-Central | Eastern-Western | Eastern-Northeastern | Central-Western | Central-Northeastern | Western-Northeastern |
| --- | --- | --- | --- | --- | --- | --- |
| 2006 | 0.2022 | 0.2603 | 0.2512 | 0.1839 | 0.1616 | 0.1764 |
| 2007 | 0.2013 | 0.2563 | 0.2459 | 0.1795 | 0.1558 | 0.1711 |
| 2008 | 0.1971 | 0.2503 | 0.2383 | 0.1735 | 0.1502 | 0.1659 |
| 2009 | 0.1955 | 0.2477 | 0.2381 | 0.1752 | 0.1547 | 0.1635 |
| 2010 | 0.1926 | 0.248 | 0.2381 | 0.1749 | 0.1552 | 0.1594 |
| 2011 | 0.1986 | 0.244 | 0.2409 | 0.1723 | 0.1572 | 0.1586 |
| 2012 | 0.1968 | 0.2465 | 0.2409 | 0.1716 | 0.1534 | 0.1582 |
| 2013 | 0.2068 | 0.2498 | 0.25 | 0.1668 | 0.156 | 0.1532 |
| 2014 | 0.2018 | 0.2434 | 0.2432 | 0.1566 | 0.1374 | 0.1543 |
| 2015 | 0.2065 | 0.2446 | 0.2433 | 0.1533 | 0.1347 | 0.1515 |
| 2016 | 0.2079 | 0.2428 | 0.2422 | 0.1509 | 0.1302 | 0.1472 |
| 2017 | 0.2136 | 0.2501 | 0.2464 | 0.1563 | 0.13 | 0.1507 |
| 2018 | 0.2149 | 0.2447 | 0.2408 | 0.1581 | 0.135 | 0.1461 |
| 2019 | 0.2114 | 0.237 | 0.2342 | 0.1472 | 0.1223 | 0.143 |
| 2020 | 0.2237 | 0.2069 | 0.2207 | 0.1393 | 0.1352 | 0.1146 |

The data for Fig 5(d)

| year | Within region | Beween regio | Intensity of transvariation |
| --- | --- | --- | --- |
| 2006 | 25.82 | 44.54 | 29.64 |
| 2007 | 25.86 | 44.13 | 30.01 |
| 2008 | 25.95 | 43.45 | 30.6 |
| 2009 | 25.79 | 44.31 | 29.91 |
| 2010 | 25.48 | 46.24 | 28.28 |
| 2011 | 25.55 | 46.45 | 28 |
| 2012 | 25.55 | 46.23 | 28.22 |
| 2013 | 25.67 | 46.79 | 27.55 |
| 2014 | 25.64 | 47.23 | 27.13 |
| 2015 | 25.99 | 45.47 | 28.54 |
| 2016 | 25.85 | 46.28 | 27.88 |
| 2017 | 25.71 | 46.44 | 27.85 |
| 2018 | 25.63 | 45.66 | 28.71 |
| 2019 | 25.86 | 44.41 | 29.73 |
| 2020 | 25.81 | 43.69 | 30.5 |

The data for Fig 7

| t | Fig 7(a) | | | | Fig 7(b) | | | Fig 7(c) | | Fig 7(d) |
| --- | --- | --- | --- | --- | --- | --- | --- | --- | --- | --- |
|  | Q1 | Q2 | Q3 | Q4 | Q1 | Q2 | Q3 | Q1 | Q2 | Q1 |
| 1 | 0.8081 | 0.7677 | 0.8344 | 0.9724 | 0.188 | 0.2117 | 0.1233 | 0.003 | 0.001 | 0.001 |
| 2 | 0.6611 | 0.6194 | 0.7414 | 0.9738 | 0.333 | 0.3686 | 0.2269 | 0.0049 | 0.0033 | 0.001 |
| 3 | 0.5323 | 0.4687 | 0.6357 | 0.9704 | 0.4508 | 0.5171 | 0.3324 | 0.0159 | 0.0094 | 0.001 |
| 4 | 0.4211 | 0.3221 | 0.5217 | 0.9695 | 0.5405 | 0.6572 | 0.4482 | 0.0374 | 0.0168 | 0.001 |
| 5 | 0.3278 | 0.2179 | 0.4277 | 0.9701 | 0.5773 | 0.7388 | 0.5527 | 0.0928 | 0.0404 | 0.0021 |
| 6 | 0.2497 | 0.1499 | 0.3486 | 0.9692 | 0.577 | 0.7661 | 0.6399 | 0.1679 | 0.084 | 0.0053 |
| 7 | 0.1872 | 0.0989 | 0.2486 | 0.9838 | 0.5529 | 0.7537 | 0.7458 | 0.2511 | 0.1474 | 0.0088 |
| 8 | 0.1277 | 0.0761 | 0.1759 | 0.9867 | 0.526 | 0.7043 | 0.8207 | 0.3345 | 0.2196 | 0.0118 |
| 9 | 0.096 | 0.0476 | 0.1013 | 0.9875 | 0.5019 | 0.6534 | 0.8987 | 0.3931 | 0.2989 | 0.009 |
| 10 | 0.0661 | 0.0239 | 0.0879 | 0.9891 | 0.4555 | 0.5529 | 0.9121 | 0.4655 | 0.4232 | 0.0129 |
