# Supplementary material for: Performance evaluation of inclusive green growth in China: Dynamic evolution, regional differences, and spatial correlation
Source: PLoS One. 2024 Jul 25;19(7):e0305338. doi: 10.1371/journal.pone.0305338 (PMC11271904; doi:10.1371/journal.pone.0305338)
Supplement: Attachment — Submitted filename: Response to Reviewers.pdf [file pone.0305338.s003.pdf]

Performance Evaluation of Inclusive Green Growth in China: Dynamic Evolution, Regional Differences, and Spatial Correlation

Manuscript ID: PONE-D-24-03913

Title: Performance Evaluation of Inclusive Green Growth in China: Dynamic Evolution, Regional Differences, and Spatial Correlation

Corresponding author: Yingchao Xu

Dear Editor and Reviewers:

We are glad to receive the feedback of our manuscript entitled "Performance Evaluation of Inclusive Green Growth in China: Dynamic Evolution, Regional Differences, and Spatial Correlation". We want to show our great appreciation to both editor and reviewers for their valuable suggestions and comments. The manuscript has been carefully revised according to reviewer's comments.

In the Response Letter, the comments are highlighted in blue, followed by point-to-point responses to each one. We sincerely hope the revised manuscript will be accepted and can be published in PLOS ONE.

Yours sincerely,

Yingchao Xu

Comments from the Reviewer # 1

The reviewer believes that the topic Performance Evaluation of Inclusive Green Growth in China: Dynamic Evolution, Regional Differences, and Spatial Correlation is worthy of investigation. However, the following needs to be addressed. There are minor and major issues that should be corrected. I believe the paper could be further strengthened by added information about.

1. Please reorganize the manuscript at the journal request. Please change the reference format.

2. The language of this manuscript is very bad and needs help from native speakers.

3. The title of the manuscript should fully demonstrate the content of this study and the relevant subjects.

4. Abstracts should include the purpose and findings of the study.

5. Introduction. This a very vague statement. These sentences do not provide any information on how the concept could be conceptualized? This section should explain the study's context and research objective. Furthermore, the research gap needs to be narrowed after analyzing the previous studies. The research method is not adequately explained in the first section.

6. Introduction, what authors wanted to convey. Here author must build research gap following the previous studies. The manuscript does not answer the following concerns: Why is it timeliness to explore such a study? What makes this study different from the previously published studies? Are there any similarly findings in line with the previously published studies? Are the findings different from prior academic studies that were conducted elsewhere, if any? For example, information innovation and innovation network, what it requires, what are the new technologies, some recent issue highlights the importance. See the following: Digital green value co-creation behavior, digital green network embedding and digital green innovation performance: moderating effects of digital green network fragmentation. Enhancing digital innovation for the sustainable transformation of manufacturing industry: a pressure-state-response system framework to perceptions of digital green innovation and its performance for green and intelligent manufacturing. Developing a Conceptual Partner Selection Framework: Digital Green Innovation Management of Prefabricated Construction Enterprises for Sustainable Urban Development.

7. Methodology: Model. I suggest authors here build your main heading on Research and data methodology. Clearly explain the model building process, and what previous studies have used similar models (model testing approach).

8. There is no flow in the text. It partly depends on the lack of proofreading but also on the fact that many statements and claims are made without being followed up by a clear and logical discussion. It is especially problematic in the Introduction that brings up a number of findings from different areas without linking them together.

9. Please make sure your conclusions' section underscores the scientific value-added of your paper, and/or the applicability of your findings/results. Highlight the novelty of your study. In addition to summarizing the actions taken and results, please strengthen the explanation of their significance. It is recommended to use quantitative reasoning comparing with appropriate benchmarks, especially those stemming from previous work. See the following:Developing a Conceptual Partner Matching Framework for Digital Green Innovation of Agricultural High-End

Equipment Manufacturing System Toward Agriculture 5.0: A Novel Niche Field Model Combined With Fuzzy VIKOR

10. More importantly, the choice of the variables should be explained in light of the theory and the prior literature on the topic. The arguments are simply relationships and causes very close to the replication of many studies dealing with the same thing. The authors should emphasize the important role of digital technology in industrial structure upgrading in future research. Some recent issue highlights the importance: The Interaction Mechanism and Dynamic Evolution of Digital Green Innovation in the Integrated Green Building Supply Chain. Please consider this structure for manuscript final part.

-Discussion

-Conclusion

-Managerial Implication

-Practical/Social Implications

11. Discussion needs to be a coherent and cohesive set of arguments that take us beyond this study in particular, and help us see the relevance of what authors have proposed. Authors should create an independent Discussion section. Author need to contextualize the findings in the literature, and need to be explicit about the added value of your study towards that literature. Also other studies should be cited to increase the theoretical background of each of the method used. Findings should be contextualized in the literature and should be explicit about the added value of the study towards the literature (New Energy-Driven Construction Industry: Digital Green Innovation Investment Project Selection of Photovoltaic Building Materials Enterprises Using an Integrated Fuzzy Decision Approach). Limitations and future research. As any emprical study that use different approaches I would like to ask to introduce in the Conclusion section at least a paragraph containing the study limitations. I noticed some things in the paper but a synthesis of statements related to how the study is useful (or partially useful, since are required certain further analysis) and helps potential interested readers does not really exist. Maybe in addition to the last section of Conclusion it is beneficial to introduce a section called: Discussion.

Responses to the Reviewer #1's Comments

Thanks very much for your careful review of our paper. These comments are very insightful and helpful. We will respond to your comments point by point below.

Comment 1: Please reorganize the manuscript at the journal request. Please change the reference format.

Response: We are grateful for your suggestion to enhance the formatting of our manuscript. We have carefully reviewed and revised our manuscript in accordance with the PLOS ONE formatting guidelines, and employ LaTeX to compose the manuscript to ensure a higher degree of standardization and clarity.

Comment 2: The language of this manuscript is very bad and needs help from native speakers.

Response: We are sincerely grateful for your attention to this matter. In an effort to elevate

the quality of our manuscript, we have polished the language throughout our manuscript. This linguistic refinement has significantly improved the clarity and fluidity of our manuscript.

Comment 3: The title of the manuscript should fully demonstrate the content of this study and the relevant subjects.

Response: Thank you very much for this question. The title "Performance Evaluation of Inclusive Green Growth in China: Dynamic Evolution, Regional Differences, and Spatial Correlation" accurately encapsulates the core focus and breadth of our research content. To provide a clear alignment between our title and the research presented within the article, we have ensured that our study addresses the following aspects: Construct an evaluation index system for inclusive green growth performance levels (IGGPL) in Chinese cities, and explore the temporal and spatial evolutions, distribution dynamics, regional disparities, and spatial correlations of IGGPLs at the city level.

Comment 4: Abstracts should include the purpose and findings of the study.

Response: Thank you very much for your suggestion. The abstract in this revised manuscript has added the purposes and findings of the study. For your convenience, we show them below.

The purposes of the study are to construct an index system for inclusive green growth performance levels (IGGPLs) in Chinese cities, measure the IGGPLs of 271 cities in the Chinese mainland based on this index system, and investigate distribution evolution, regional disparities, spatial correlations, and state transition of IGGPLs at the city level.

The research findings reveal that: (1) China's IGGPL has improved rapidly, but regions with lower IGGPLs still predominate. The eastern region is far ahead, followed by the northeastern region, with the western and center regions trailing; (2) The development trends of IGGPLs in the eastern and central regions are positive, with no signs of polarization. Although polarizations are obvious in the western and northeastern but have been improved significantly; (3) Regional differences exist, but are gradually narrowing over time. By decomposing regional differences, we find that regional differences are the main cause of total differences; (4) the IGGPLs of cities have significant spatial correlations, presenting the spatial agglomeration characteristics of 'high-high' and 'low-low'.

Comment 5: Introduction. This a very vague statement. These sentences do not provide any information on how the concept could be conceptualized? This section should explain the study's context and research objective. Furthermore, the research gap needs to be narrowed after analyzing the previous studies. The research method is not adequately explained in the first section.

Response: Thank you very much for these questions. The statements of the introduction in this revised manuscript have been polished from the perspectives of research contexts, research objectives, the research gaps of previous studies, and research methods. For your convenience, we show them below.

The research contexts of this manuscript are as follows. In recent years, with the rapid development of industrialization and urbanization, the living environment of mankind has been dramatically improved, the material foundations have become more abundant, and billions of people

have shaken off poverty. However, these economic prosperities lie in the sacrifice of the environment (Zhong et al., 2021 [12]). In the past, the development patterns of high investment, high consumption, and high pollution have led to many severe ecological problems, such as the depletion of natural resources, air pollution, and the destruction of biodiversity (Fan et al., 2023 [2]; Ghafarpasand et al., 2021 [3]; Kassouri, 2021 [5]). In addition, unbalanced regional development has caused many social problems, such as regional development gaps, wealth gaps, and uneven development opportunities (Sulemana et al., 2019 [10]). In this context, the concept of inclusive green growth was first proposed at the Rio+20 Summit in 2012. Subsequently, the World Bank formally proposed this concept, emphasizing that industrial economic growth must consider inclusiveness and greenness. This concept offers a new development direction for many developing countries and has received widespread attention in the academic community.

The research objectives of this manuscript are as follows. This article aims to construct an evaluation index system for IGGPL in Chinese cities and to utilize panel data from 271 Chinese cities from 2006 to 2020 to explore the temporal and spatial evolutions, distribution dynamics, regional disparities, and spatial correlations of IGGPLs at the city level. The marginal contributions of this article are as follows. First, according to the unique economic-social-environmental characteristics of Chinese cities, we design an evaluation index system for inclusive green growth at the prefecture-level city that considers China's conditions, enriching the theory of measuring IGGPL. Second, we provide comprehensive analyses of the temporal and spatial evolutions, distribution dynamics, and regional disparities of the IGGPLs at the city level in China by using a variety of statistical methods. Third, we investigate the transition probabilities of IGGPLs, uncovering the intrinsic reasons behind the formation of the evolutionary characteristics of IGGPLs in Chinese cities.

The existing research gaps of previous studies are as follows. In terms of the concept of inclusive green growth, the academic community has not reached a consensus on the concept of inclusive green growth. According to the existing studies on the concept of inclusive green growth, this paper believes that inclusive green growth is a balanced growth pattern that aims to enhance the welfare of both current and future generations and achieves stable economic growth under the conditions of minimizing the consumption of natural assets and maximizing social equity, ultimately promoting sustainable development. In addition, due to differences in national backgrounds, population status, resource endowments, and institution designs among different countries, the strategies for promoting inclusive green growth vary from country to country. As the largest developing country, China is undergoing the crucial stage of economic structural adjustment, and many deep-seated issues in the traditional economic developments have gradually emerged, leading to problems such as unbalanced and inadequate economic development. However, research on China's IGGPL is mainly focused on provinces, economic zones, or city clusters, with fewer studies exploring the evolution characteristics of IGGPL at prefecture-level cities. There is a lack of a relatively objective evaluation index system for assessing the IGGPLs of cities in China, and fewer studies have conducted in-depth analyses of the spatial correlations and spatial agglomeration characteristics of China's city-level IGGPLs. Moreover, scholars have

rarely offered the underlying reasons behind the evolutionary characteristics of China's IGGPL from a quantitative perspective.

The research methods of this manuscript are as follows. This article provides multifaceted analyses of the temporal and spatial evolutions, distribution dynamics, and regional disparities of the IGGPLs at the city level in China by using a variety of statistical methods, including the VHS-D method, Kernel density method, Dagum-Gini coefficient method, Moran index method, and Markov chain method. Our methods ensure that the findings are statistically sound and provide a reliable basis for future research directions.

Comment 6: Introduction, what authors wanted to convey. Here author must build research gap following the previous studies. The manuscript does not answer the following concerns: Why is it timeliness to explore such a study? What makes this study different from the previously published studies? Are there any similarly findings in line with the previously published studies? Are the findings different from prior academic studies that were conducted elsewhere, if any? For example, information innovation and innovation network, what it requires, what are the new technologies, some recent issue highlights the importance. See the following: Digital green value co-creation behavior, digital green network embedding and digital green innovation performance: moderating effects of digital green network fragmentation. Enhancing digital innovation for the sustainable transformation of manufacturing industry: a pressure-state-response system framework to perceptions of digital green innovation and its performance for green and intelligent manufacturing. Developing a Conceptual Partner Selection Framework: Digital Green Innovation Management of Prefabricated Construction Enterprises for Sustainable Urban Development.

Response: Thank you very much for these questions. In our revised manuscript, we have pointed out the research gaps left by previous studies, emphasizing the timeliness and uniqueness of our exploration. Here's a detailed elucidation of these aspects within the introduction.

We provide the following response, explaining why exploring such a study is timely and how it differs from previously published studies. As the largest developing country, China is undergoing the crucial stage of economic structural adjustment, and many deep-seated issues in the traditional economic developments have gradually emerged, leading to problems such as unbalanced and inadequate economic development. However, research on China's IGGPL is mainly focused on provinces, economic zones, or city clusters, with fewer studies exploring the evolution characteristics of IGGPL at prefecture-level cities. There is a lack of a relatively objective evaluation index system for assessing the IGGPLs of cities in China, and fewer studies have conducted in-depth analyses of the spatial correlations and spatial agglomeration characteristics of China's city-level IGGPLs. Moreover, scholars have rarely offered the underlying reasons behind the evolutionary characteristics of China's IGGPL from a quantitative perspective.

Regarding the question of whether there are similar findings consistent with previously published studies, we provide the following responses. The existing research on China's IGGPL is mainly focused on provinces, economic zones, or city clusters, with fewer studies exploring the evolution characteristics of IGGPL at prefecture-level cities. There is a lack of a relatively objec-

tive evaluation index system for assessing the IGGPLs of cities in China, and fewer studies have conducted in-depth analyses of the spatial correlations and spatial agglomeration characteristics of China's city-level IGGPLs. Moreover, scholars have rarely offered the underlying reasons behind the evolutionary characteristics of China's IGGPL from a quantitative perspective. Therefore, it is not possible to compare our findings in parallel with those of existing studies.

Comment 7: Methodology: Model. I suggest authors here build your main heading on Research and data methodology. Clearly explain the model building process, and what previous studies have used similar models (model testing approach)

Response: I appreciate your suggestions greatly. This revised manuscript has built the main headings on research and data methodology, providing a detailed explanation of the methods used (including the vertical and horizontal scatter degree method, Kernel density method, Dagum-Gini coefficient method, Moran index method, and Markov chain method).

Comment 8: There is no flow in the text. It partly depends on the lack of proofreading but also on the fact that many statements and claims are made without being followed up by a clear and logical discussion. It is especially problematic in the Introduction that brings up a number of findings from different areas without linking them together.

Response: Thank you very much for this question. In this revised manuscript, we have endeavored to refine the introduction. Within it, we summarized some findings from different fields and successfully linked these findings.

Comment 9: Please make sure your conclusions' section underscores the scientific value-added of your paper, and/or the applicability of your findings or results. Highlight the novelty of your study. In addition to summarizing the actions taken and results, please strengthen the explanation of their significance. It is recommended to use quantitative reasoning comparing with appropriate benchmarks, especially those stemming from previous work. See the following: Developing a Conceptual Partner Matching Framework for Digital Green Innovation of Agricultural High-End Equipment Manufacturing System Toward Agriculture 5.0: A Novel Niche Field Model Combined With Fuzzy VIKOR.

Response: Thank you very much for these suggestions. The conclusions in this manuscript have been polished. For your convenience, we show them below.

The scientific value-added, and the applicability of our findings of this study are as follows. This article establishes an evaluation index system for IGGPL, and calculates the IGGPLs in 271 cities across China from 2006 to 2020 based on the index system. We analyze the temporal and spatial evolutions, regional disparities, and spatial correlations of China's IGGPLs. The findings of this study can provide insights for the effectiveness evaluation of China's inclusive green growth strategy and related research on sustainable development. In addition, the research method and ideas of this paper have a certain universality, and the following aspects can be further studied in the future. Firstly, we can take the county-level IGGPL in China as the research subject, explore the evolutionary characteristics of the county-level IGGPLs, and thus establish the evaluation index systems of IGGPLs in China across "national-economic belt-province-city-county" levels. In addition, we can compare the IGGPLs of major cities in China with those in other countries by

constructing an index system capable of international comparisons.

Regarding the issue of using quantitative reasoning to compare with appropriate benchmarks, especially those from previous work, we provide the following responses. The existing research on China's IGGPL is mainly focused on provinces, economic zones, or city clusters, with fewer studies exploring the evolution characteristics of IGGPL at prefecture-level cities. There is a lack of a relatively objective evaluation index system for assessing the IGGPLs of cities in China, and fewer studies have conducted in-depth analyses of the spatial correlations and spatial agglomeration characteristics of China's city-level IGGPLs. Moreover, scholars have rarely offered the underlying reasons behind the evolutionary characteristics of China's IGGPL from a quantitative perspective. Therefore, it is not possible to compare our findings in parallel with those of existing studies.

Comment 10: More importantly, the choice of the variables should be explained in light of the theory and the prior literature on the topic. The arguments are simply relationships and causes very close to the replication of many studies dealing with the same thing. The authors should emphasize the important role of digital technology in industrial structure upgrading in future research. Some recent issue highlights the importance: The Interaction Mechanism and Dynamic Evolution of Digital Green Innovation in the Integrated Green Building Supply Chain. Please consider this structure for manuscript final part. -Discussion -Conclusion -Managerial Implication -Practical/Social Implications.

Response: Thank you very much for these suggestions. The choice of the variables was not explained in our original manuscript, and we are very sorry about this. This revised manuscript has referred to "The Interaction Mechanism and Dynamic Evolution of Digital Green Innovation in the Integrated Green Building Supply Chain" and added the literature for variable selection in the indicator system.

Comment 11: Discussion needs to be a coherent and cohesive set of arguments that take us beyond this study in particular, and help us see the relevance of what authors have proposed. Authors should create an independent Discussion section. Author need to contextualize the findings in the literature, and need to be explicit about the added value of your study towards that literature. Also other studies should be cited to increase the theoretical background of each of the method used. Findings should be contextualized in the literature and should be explicit about the added value of the study towards the literature (New Energy-Driven Construction Industry: Digital Green Innovation Investment Project Selection of Photovoltaic Building Materials Enterprises Using an Integrated Fuzzy Decision Approach). Limitations and future research. As any empirical study that use different approaches I would like to ask to introduce in the Conclusion section at least a paragraph containing the study limitations. I noticed some things in the paper but a synthesis of statements related to how the study is useful (or partially useful, since are required certain further analysis) and helps potential interested readers does not really exist. Maybe in addition to the last section of Conclusion it is beneficial to introduce a section called: Discussion.

Response: Thank you very much for these suggestions. The statements of the introduction in

this manuscript have been polished according to your valuable suggestions. In addition, we have referred to the “New Energy-Driven Construction Industry: Digital Green Innovation Investment Project Selection of Photovoltaic Building Materials Enterprises Using an Integrated Fuzzy Decision Approach” and added the limitations of this study and future research in the conclusion and discussion section of the revised manuscript. For your convenience, we show them below.

This study has certain limitations. We refer to a large body of related research and follow strict procedures in constructing the index system of IGGPL, and thus measure the IGGPL in China. However, due to data limitations, some indicators are not included in this index system. There is room for improving the index system of IGGPL. The research method and ideas of this paper have a certain universality, and the following aspects can be further studied in the future. Firstly, we can take the county-level IGGPL in China as the research subject, explore the evolutionary characteristics of the county-level IGGPLs, and thus establish the evaluation index systems of IGGPLs in China across “national - economic belt - province - city - county” levels. In addition, we can compare the IGGPLs of major cities in China with those in other countries by constructing an index system capable of international comparisons.

Comments from the Reviewer # 2

Inclusive green growth is an essential approach to achieving sustainable development. This manuscript constructs a system of IGGPLs indicators from using city panel data, further using the methods of vertical and horizontal scatter degree and linear synthesis. Spatial correlation models, regional difference decomposition models, and spatial state transition models are employed to investigate the spatial distribution characteristics and dynamic evolution trends of IGGPLs. Of particular interest in this manuscript is the construction of an evaluation indicator system for IGGPLs from the city level, and the measurement and evaluation of IGGPLs in China’s 271 cities, which is a relatively novel perspective, as most studies have focused on the macro-administrative scale of provinces. In contrast, further refinement of the evaluation index system can provide specific guidance for the green transformation of the economy. But before accepting publication, there are still some elements that need further explanation which will be more conducive to improving the quality of the manuscript:

(1) The introduction section does not adequately clarify the scientific issues. It is not stated why IGGPLs are analysed from a spatial perspective and it is recommended that the authors add this section.

(2) The W matrix used by the authors is the 0-1 neighborhood matrix. Would the authors please give full consideration to whether this matrix is sufficiently interpretable and whether a geographic distance matrix, an economic distance matrix, or an economic-geographic nested matrix would be more applicable?

(3) In the results and analyses section, the authors provide a lot of descriptions around the empirical results, but the reasons for the results are not fully elaborated. It is recommended that the authors not only present the empirical results, but also analyse more around the real situation, which may be conducive to a more focused opinion.

In summary, I suggest that the author make minor revisions to the manuscript.

Responses to the Reviewer #2's Comments

Thanks very much for your careful review of our paper. These comments are very insightful and helpful. We will respond to your comments point by point below.

Comment 1: The introduction section does not adequately clarify the scientific issues. It is not stated why IGGPLs are analyzed from a spatial perspective and it is recommended that the authors add this section.

Response: Thank you very much for pointing out this question. The reason why this article explores the spatial correlation of IGGPL from a spatial perspective is that we have found that more literature has discussed the regional differences of IGGPL, but these regional differences of IGGPL can lead to spatial agglomerations in a local range. According to the first law of geography, geographical objects or attributes are interrelated in spatial distribution. This article explores from this perspective, filling the gaps in existing research.

Comment 2: The W matrix used by the authors is the 0-1 neighborhood matrix. Would the authors please give full consideration to whether this matrix is sufficiently interpretable and whether a geographic distance matrix, an economic distance matrix, or an economic-geographic nested matrix would be more applicable?

Response: Thank you very much for this question. We use a 0-1 spatial weight matrix because we believe that two adjacent cities are closer and have closer connections with each other, resulting in a stronger correlation. We also used spatial weight matrices based on longitude and latitude calculations and economic-geographic nested spatial weight matrices, and the conclusions obtained are roughly similar, indicating that the 0-1 spatial weight matrix is robust.

Comment 3: In the results and analyses section, the authors provide a lot of descriptions around the empirical results, but the reasons for the results are not fully elaborated. It is recommended that the authors not only present the empirical results, but also analyse more around the real situation, which may be conducive to a more focused opinion.

Response: Thank you very much for these suggestions. We have provided the more detailed explanations of the reasons underlying our empirical research results in this revised manuscript. For your convenience, we give some examples to show them below.

(i) The explanation for this phenomenon is that in November 2012, the Chinese government issued the policy "Guiding Opinions on Accelerating Green Development and Building a Beautiful China". Under the guidance of this policy, governments at all levels in China actively promote the optimization and upgrading of industrial structures. In the process of economic development, they not only focus on environmental protection and resource conservation but also attach importance to improving people's livelihoods and reducing poverty, which has positive impacts on the environment and society

(ii) Based on the analyses above, it is evident that there are differences in IGGPLs among different cities in China, i.e., total differences, and these differences gradually decrease over time. The total differences can be divided into three parts: intra-regional differences, inter-regional net

difference differences, and the intensity of trans-differences. Among them, the inter-regional differences are the main reasons for the total differences, and the development gaps between the eastern region and the other three major regions are the key factors causing inter-regional differences. The reasons behind these are that the eastern region has earlier industrial development, faster economic growth, more complete infrastructure and public services, and more investment in social and environmental governance, which can attract a large influx of population, talent, technology, resources, and funds from the central, western, and northeastern regions. These led to faster development in IGGPLs in the eastern region than in the other three regions.

Comments from the Reviewer # 3

Review comments on "Performance Evaluation of Inclusive Green Growth in China: Dynamic Evolution, Regional Differences, and Spatial Correlation" This research construct an index system for inclusive green growth performance levels (IGGPLs) in Chinese cities. The paper has some fallouts.

1. In the introduction, the overview of the existing research needs to be improved, and the elaboration of the research necessity is insufficient, and does not introduce the policy background of China's inclusive green growth performance levels (IGGPLs).

2. In section 2.1, this part of literature review only reviews "inclusive growth" and "green growth" respectively, and the authors lack in-depth thinking about the concept of inclusive green growth performance levels (IGGPLs).

3. In section 2.2, this part of literature review contains a lot of content and needs to sort out the logic. It is recommended to add a table to make the content clearer.

4. section 2.3 is not sufficient for the review of existing literature, so it can be considered to be combined into section 2.1 and section 2.2, and the major contributions can be considered to be reflected in the introduction and the theoretical contributions at the end.

5. The theoretical basis for the construction of section 3.1 and section 3.2 index system is insufficient, and the reference of relevant literatures is lacking.

6.section 3.3, "and the data after 2020, due to the pandemic, has stronger fluctuations and more outliers" proposes to delete this sentence, Large fluctuations in the data should not be used as a basis for narrowing the scope of the study.

7. It is suggested that authors refer to PLOS ONE format to further standardize the format of this paper.

In summary, it is recommended a minor revision of the manuscript.

Responses to the Reviewer #3's Comments

Comment 1: In the introduction, the overview of the existing research needs to be improved, and the elaboration of the research necessity is insufficient, and does not introduce the policy background of China's inclusive green growth performance levels (IGGPLs).

Response: Thank you very much for your suggestion. In the introduction, we have improved the overview of existing research, further elaborated on the necessity of research, and supplemented the policy background of China's inclusive green growth performance level.

To improve the overview of existing research, we have reorganized literature to make the content clearer. Based on the research content of this article, we adopt the approach of first introducing the concept studies of inclusive green growth, then the measuring studies of the inclusive green growth performance level (IGGPL), and finally the studies on the factors affecting IGGPL for literature review. In terms of the concept studies of inclusive green growth, we discuss two schools of thought. One emphasizes the importance of greenness for inclusive green growth from the perspective of development economics, and the other school of thought emphasizes the importance of inclusiveness for inclusive green growth in terms of welfare economics. However, the academic community has not reached a consensus on the concept of inclusive green growth. Regarding the research on measuring IGGPL, the academic community has mainly developed two types of literature, i.e., exploring IGGPL from the perspective of methodological improvement and constructing evaluation indicator systems. In addition, existing studies on the impact factors of IGGPL mainly focus on the economy and policy factors.

The necessities of research are as follows. Due to differences in national backgrounds, population status, resource endowments, and institution designs among different countries, the strategies for promoting inclusive green growth vary from country to country. As the largest developing country, China is undergoing the crucial stage of economic structural adjustment, and many deep-seated issues in the traditional economic developments have gradually emerged, leading to problems such as unbalanced and inadequate economic development. However, research on China's IGGPL is mainly focused on provinces, economic zones, or city clusters, with fewer studies exploring the evolution characteristics of IGGPL at prefecture-level cities. There is a lack of a relatively objective evaluation index system for assessing the IGGPLs of cities in China, and fewer studies have conducted in-depth analyses of the spatial correlations and spatial agglomeration characteristics of China's city-level IGGPLs. Moreover, scholars have rarely offered the underlying reasons behind the evolutionary characteristics of China's IGGPL from a quantitative perspective.

This article takes IGGPL as the research object, which is in line with China's current goals of achieving peak carbon emissions and carbon neutrality.

Comment 2: In section 2.1, this part of literature review only reviews "inclusive growth" and "green growth" respectively, and the authors lack in-depth thinking about the concept of inclusive green growth performance levels (IGGPLs)

Response: Thank you very much for your suggestion. We have supplemented the concept of IGGPL in this revised manuscript. According to the existing studies on the inclusive green growth concept, this paper believes that inclusive green growth is a balanced growth pattern that aims to enhance the welfare of both current and future generations and achieves stable economic growth under the conditions of minimizing the consumption of natural assets and maximizing social equity, ultimately promoting sustainable development.

In addition, we also provided the following three characteristics of IGGPL. One is the economic growth (Ali and Zhuang, 2007 [1]; Sugden, 2012 [9]). Economic growth is the starting point for inclusive green growth. The sustainable development of human beings cannot be achieved without

solid material bases. Only sustainable economic growth can create more social wealth and employment opportunities, thus providing a solid material guarantee for the inclusive development of human society. The second is the social inclusion (Spanish, 2021 [7]; Quaas and Smulders, 2018 [6]; Hu and Wang, 2019 [4]). Through the promotion of fair opportunities for members of society in such areas as employment, health care, education, social security, and public resources, the growing problem of social inequality will be resolved so that people can equally participate in the development process and enjoy the fruits of development, thereby ultimately realizing the goal of sustainable development. The third is the environmental greening (Wellmann et al., 2020 [11]; Spratt, 2013 [8]). Environmental greening is a foundation of inclusive green growth. The realization of sustainable development needs to fully consider the coordinated development of human beings and nature so that limited natural assets can continuously serve human development. By enlarging green ecological space, relaxing environmental pressure, and strengthening the management of pollution emissions, the negative impacts of human production activities on the ecological environment will be effectively mitigated, the over-exploitation of natural resources will be avoided, and natural assets will be able to provide human beings with the resources and environment on which they depend in the long term, thereby ultimately realizing sustainable development.

Comment 3: In section 2.2, this part of literature review contains a lot of content and needs to sort out the logic. It is recommended to add a table to make the content clearer.

Response: Thank you very much for your valuable suggestion. In this revised manuscript, we have reorganized literature to make the content clearer. Based on the research content of this article, we adopt the approach of first introducing the concept studies of inclusive green growth, then the measuring studies of the inclusive green growth performance level (IGGPL), and finally the studies on the factors affecting IGGPL for literature review. In terms of the concept studies of inclusive green growth, we discuss two schools of thought. One emphasizes the importance of greenness for inclusive green growth from the perspective of development economics, and the other school of thought emphasizes the importance of inclusiveness for inclusive green growth in terms of welfare economics. However, the academic community has not reached a consensus on the concept of inclusive green growth. Regarding the research on measuring IGGPL, the academic community has mainly developed two types of literature, i.e., exploring IGGPL from the perspective of methodological improvement and constructing evaluation indicator systems. In addition, existing studies on the impact factors of IGGPL mainly focus on the economy and policy factors.

Comment 4: Section 2.3 is not sufficient for the review of existing literature, so it can be considered to be combined into section 2.1 and section 2.2, and the major contributions can be considered to be reflected in the introduction and the theoretical contributions at the end.

Response: We are truly grateful for your constructive suggestion. In this revised draft, we have meticulously reorganized the literature reviews of our manuscript to ensure that the major contributions of our study are now reflected at the end of the introduction.

Comment 5: The theoretical basis for the construction of section 3.1 and section 3.2 index system is insufficient, and the reference of relevant literatures is lacking.

Response: We extend our gratitude for your insightful suggestion. Recognizing the importance

of a solid theoretical foundation for our index system, we have taken steps to rectify the oversight in our initial submission. In this revised manuscript, we have incorporated the robust explanations of the theoretical framework that underpins our selection of variables. At the same time, in the descriptions of the characteristics of IGGPL, the relevant literature has also been supplemented.

Comment 6: Section 3.3, "and the data after 2020, due to the pandemic, has stronger fluctuations and more outliers" proposes to delete this sentence, Large fluctuations in the data should not be used as a basis for narrowing the scope of the study.

Response: We appreciate your suggestion, and in response, we have carefully reviewed the relative content of our manuscript. As a result of this review, we have decided to delete this sentence.

Comment 7: It is suggested that authors refer to PLOS ONE format to further standardize the format of this paper.

Response: We are grateful for your suggestion to enhance the formatting of our manuscript. We have carefully reviewed and revised our manuscript in accordance with the PLOS ONE formatting guidelines, and employ LaTeX to compose the manuscript to ensure a higher degree of standardization and clarity.

Comments from the Reviewer # 4

1. The description of the article's research purpose or research contribution/innovation in the introduction is not clear and unambiguous. Why this study is necessary? What policy level problem this study is addressing? How the study is expected to provide any solution to that problem? How does the choice of sample is complementing that problem? Are the results and policies generalizable? The introduction is silent in all these aspects.

2. The article is presented only as an expression of results and lacks in-depth explanation of the reasons behind the results.

3. The literature review is not enough, the contribution made by previous studies has not been clearly expressed, and the author needs to introduce the research methods of previous articles to highlight the innovation of this paper.

4. There are also some grammatical errors formatting errors in the sentence expressions, so please check them carefully.

5. Important parts of the discussion are missing. Authors should focus on describing the differences between the article's research and that of other scholars, thus highlighting the article's relevance and academic value.

6. The article was not written following the correct journal's guidelines to be considered for publication. INTORDCUTION → MRTHOD → RESULTS → DISSCUSION → CONCLUSION.

Responses to the Reviewer #4's Comments

Comment 1: The description of the article's research purpose or research contribution or innovation in the introduction is not clear and unambiguous. Why this study is necessary? What policy level problem this study is addressing? How is the study expected to provide any

solution to that problem? How does the choice of sample is complementing that problem? Are the results and policies generalizable? The introduction is silent in all these aspects.

Response: Thank you very much for these questions. The introduction in this revised manuscript has clearly described the research purpose, research contribution (or innovation), the necessity of this study, the policy-level problem that needs to be addressed, the solution provided for this policy-level problem, the choice of sample, and the question of whether the results and policies are generalizable. For your convenience, we show them below.

The research purposes of this manuscript are as follows. This article aims to construct an evaluation index system for IGGPL in Chinese cities and to utilize panel data from 271 Chinese cities from 2006 to 2020 to explore the temporal and spatial evolutions, distribution dynamics, regional disparities, and spatial correlations of IGGPLs at the city level. The marginal contributions of this article are as follows. First, according to the unique economic-social-environmental characteristics of Chinese cities, we design an evaluation index system for inclusive green growth at the prefecture-level city that considers China's conditions, enriching the theory of measuring IGGPL. Second, we provide comprehensive analyses of the temporal and spatial evolutions, distribution dynamics, and regional disparities of the IGGPLs at the city level in China by using a variety of statistical methods. Third, we investigate the transition probabilities of IGGPLs, uncovering the intrinsic reasons behind the formation of the evolutionary characteristics of IGGPLs in Chinese cities.

The marginal contributions of this article are as follows. First, according to the unique economic - social-environmental characteristics of Chinese cities, we design an evaluation index system for inclusive green growth at the prefecture-level city that considers China's conditions, enriching the theory of measuring IGGPL and being in line with China's current goals of achieving peak carbon emissions and carbon neutrality. Second, we provide comprehensive analyses of the temporal and spatial evolutions, distribution dynamics, and regional disparities of the IGGPLs at the city level in China by using a variety of statistical methods. Third, we investigate the transition probabilities of IGGPLs, uncovering the intrinsic reasons behind the formation of the evolutionary characteristics of IGGPLs in Chinese cities.

The necessities of this study are as follows. In terms of the concept of inclusive green growth, the academic community has not reached a consensus on the concept of inclusive green growth. According to the existing studies on the concept of inclusive green growth, this paper believes that inclusive green growth is a balanced growth pattern that aims to enhance the welfare of both current and future generations and achieves stable economic growth under the conditions of minimizing the consumption of natural assets and maximizing social equity, ultimately promoting sustainable development. In addition, due to differences in national backgrounds, population status, resource endowments, and institution designs among different countries, the strategies for promoting inclusive green growth vary from country to country. As the largest developing country, China is undergoing the crucial stage of economic structural adjustment, and many deep-seated issues in the traditional economic developments have gradually emerged, leading to problems such as unbalanced and inadequate economic development. However, research on China's IGGPL is

mainly focused on provinces, economic zones, or city clusters, with fewer studies exploring the evolution characteristics of IGGPL at prefecture-level cities. There is a lack of a relatively objective evaluation index system for assessing the IGGPLs of cities in China, and fewer studies have conducted in-depth analyses of the spatial correlations and spatial agglomeration characteristics of China's city-level IGGPLs. Moreover, scholars have rarely offered the underlying reasons behind the evolutionary characteristics of China's IGGPL from a quantitative perspective.

The policy-level problem that needs to be addressed are as follows. This article takes IGGPL as the research object, which is in line with China's current goals of achieving peak carbon emissions and carbon neutrality. In order to promote the implementation of this policy, it is necessary to encourage enterprises to increase investment in research and development of energy-saving and emission reduction technologies, use new energy, clean energy and renewable energy, promote the green transformation of production mode of enterpris es, and reduce per capita energy consumption and pollution per unit output value.

Regarding the selection of samples and the generalizability of results and policies, our responses are as follows. We investigate the status of China's IGGPL at the urban level, as the ultimate policy objective of achieving peak carbon emissions and carbon neutrality in China is set at the city scale. Hence, choosing samples at this level is both practical and valuable. Moreover, the sample cities we selected represent over 93% of all cities in China, ensuring broad coverage. Therefore, the findings and insights yielded possess a certain degree of universality.

Comment 2: The article is presented only as an expression of results and lacks in-depth explanation of the reasons behind the results.

Response: Thank you very much for these questions. We have provided the more detailed explanations of the reasons underlying our empirical research results in this revised manuscript. For your convenience, we give some examples to show them below.

(i) The explanation for this phenomenon is that in November 2012, the Chinese government issued the policy "Guiding Opinions on Accelerating Green Development and Building a Beautiful China". Under the guidance of this policy, governments at all levels in China actively promote the optimization and upgrading of industrial structures. In the process of economic development, they not only focus on environmental protection and resource conservation but also attach importance to improving people's livelihoods and reducing poverty, which has positive impacts on the environment and society

(ii) Based on the analyses above, it is evident that there are differences in IGGPLs among different cities in China, i.e., total differences, and these differences gradually decrease over time. The total differences can be divided into three parts: intra-regional differences, inter-regional net difference differences, and the intensity of trans-differences. Among them, the inter-regional differences are the main reasons for the total differences, and the development gaps between the eastern region and the other three major regions are the key factors causing inter-regional differences. The reasons behind these are that the eastern region has earlier industrial development, faster economic growth, more complete infrastructure and public services, and more investment in social and environmental governance, which can attract a large influx of population, talent,

technology, resources, and funds from the central, western, and northeastern regions. These led to faster development in IGGPLs in the eastern region than in the other three regions.

Comment 3: The literature review is not enough, the contribution made by previous studies has not been clearly expressed, and the author needs to introduce the research methods of previous articles to highlight the innovation of this paper.

Response: Thank you very much for these questions. In this revised manuscript, based on the research content of this article, we adopt the approach of first introducing the concept studies of inclusive green growth, then the measuring studies of the inclusive green growth performance level (IGGPL), and finally the studies on the factors affecting IGGPL for literature review. In terms of the concept studies of inclusive green growth, we discuss two schools of thought. One emphasizes the importance of greenness for inclusive green growth from the perspective of development economics, and the other school of thought emphasizes the importance of inclusiveness for inclusive green growth in terms of welfare economics. However, the academic community has not reached a consensus on the concept of inclusive green growth. Regarding the research on measuring IGGPL, the academic community has mainly developed two types of literature, i.e., exploring IGGPL from the perspective of methodological improvement and constructing evaluation indicator systems. In addition, existing studies on the impact factors of IGGPL mainly focus on the economy and policy factors.

The existing literature has conducted a significant amount of work on the concept of inclusive green growth, the statistical measurement of IGGPL, and the factors affecting IGGPL. This research plays an important role in scientifically achieving the essence of inclusive green growth. According to the existing studies on the inclusive green growth concept, this paper believes that inclusive green growth is a balanced growth pattern that aims to enhance the welfare of both current and future generations and achieves stable economic growth under the conditions of minimizing the consumption of natural assets and maximizing social equity, ultimately promoting sustainable development. Due to differences in national backgrounds, population status, resource endowments, and institution designs among different countries, the strategies for promoting inclusive green growth vary from country to country. However, research on China's IGGPL is mainly focused on provinces, economic zones, or city clusters, with fewer studies exploring the evolution characteristics of IGGPL at prefecture-level cities. There is a lack of a relatively objective evaluation index system for assessing the IGGPLs of cities in China, and fewer studies have conducted in-depth analyses of the spatial correlations and spatial agglomeration characteristics of China's city-level IGGPLs. Moreover, scholars have rarely offered the underlying reasons behind the evolutionary characteristics of China's IGGPL from a quantitative perspective.

Comment 4: There are also some grammatical errors formatting errors in the sentence expressions, so please check them carefully.

Response: Thank you very much for the grammatical errors formatting errors you raised. We have thoroughly reviewed the manuscript and implemented the necessary corrections to enhance the clarity and professionalism of our revised submission.

Comment 5: Important parts of the discussion are missing. Authors should focus on describ-

ing the differences between the article's research and that of other scholars, thus highlighting the article's relevance and academic value.

Response: Thank you for your question. We have added the differences between our research and other scholars' research in the discussion, emphasizing the relevance and academic value of the article. For your convenience, we show them below.

Due to differences in national backgrounds, population status, resource endowments, and institution designs among different countries, the strategies for promoting inclusive green growth vary from country to country. As the largest developing country, China is undergoing the crucial stage of economic structural adjustment, and many deep-seated issues in the traditional economic developments have gradually emerged, leading to problems such as unbalanced and inadequate economic development. However, research on China's IGGPL is mainly focused on provinces, economic zones, or city clusters, with fewer studies exploring the evolution characteristics of IGGPL at prefecture-level cities. There is a lack of a relatively objective evaluation index system for assessing the IGGPLs of cities in China, and fewer studies have conducted in-depth analyses of the spatial correlations and spatial agglomeration characteristics of China's city-level IGGPLs. Moreover, scholars have rarely offered the underlying reasons behind the evolutionary characteristics of China's IGGPL from a quantitative perspective.

Given these, this article constructs an evaluation index system for IGGPL in Chinese cities and utilizes panel data from 271 Chinese cities from 2006 to 2020 to explore the temporal and spatial evolutions, distribution dynamics, regional disparities, and spatial correlations of IGGPLs at the city level. The marginal contributions of this article are as follows. First, according to the unique economic-social-environmental characteristics of Chinese cities, we design an evaluation index system for inclusive green growth at the prefecture-level city that considers China's conditions, enriching the theory of measuring IGGPL and being in line with China's current goals of achieving peak carbon emissions and carbon neutrality. Second, we provide comprehensive analyses of the temporal and spatial evolutions, distribution dynamics, and regional disparities of the IGGPLs at the city level in China by using a variety of statistical methods. Third, we investigate the transition probabilities of IGGPLs, uncovering the intrinsic reasons behind the formation of the evolutionary characteristics of IGGPLs in Chinese cities.

Comment 6: The article was not written following the correct journal's guidelines to be considered for publication. INTORDCUTION → MRTHOD → RESULTS → DISSCUSION → CONCLUSION.

Response: We are deeply thankful for your insightful suggestion. Following your guidance, we have meticulously revised the structure of our article. Notably, we integrated the literature review into the introduction, delineated a separate research methodology section, and concluded with a dedicated discussion. These modifications aim to enhance the coherence and clarity of our article's presentation.

References

[1] I Ali and J Zhuang. Inclusive growth toward a prosperous asia: Policy implications. economics and research department. 2007.

[2] Shuangshuang Fan, Hongyun Huang, William Mbanyele, Zihao Guo, and Chenxi Zhang. Inclusive green growth for sustainable development of cities in china: spatiotemporal differences and influencing factors. *Environmental Science and Pollution Research*, 30(4):11025–11045, 2023.

[3] Omid Ghaffarpasand, Mohammad Reza Talaie, Hossein Ahmadikia, Amirreza TalaieKhozani, Maryam Davari Shalamzari, and Sina Majidi. How does unsustainable urbanization affect driving behavior and vehicular emissions? evidence from iran. *Sustainable Cities and Society*, 72:103065, 2021.

[4] Wanyang Hu and Rui Wang. Which chinese cities are more inclusive and why? *Cities*, 86:51–61, 2019.

[5] Yacouba Kassouri. Monitoring the spatial spillover effects of urbanization on water, built-up land and ecological footprints in sub-saharan africa. *Journal of Environmental Management*, 300:113690, 2021.

[6] Martin F Quaas and Sjak Smulders. Brown growth, green growth, and the efficiency of urbanization. *Environmental and resource economics*, 71:529–549, 2018.

[7] Clive L Spash. the economyas if people mattered: revisiting critiques of economic growth in a time of crisis. *Globalizations*, 18(7):1087–1104, 2021.

[8] Stephen Spratt and Stephany Griffith-Jones. *Mobilising investment for inclusive green growth in low-income countries*. Deutsche Gesellschaft für Internationale Zusammenarbeit (GIZ) GmbH, 2013.

[9] Craig Sugden. Is growth in asia and the pacific inclusive? *Asian Development Bank Economics Working Paper Series*, (317), 2012.

[10] Iddisah Sulemana, Edward Nketiah-Amponsah, Emmanuel A Codjoe, and Jennifer Akua Nyarko Andoh. Urbanization and income inequality in sub-saharan africa. *Sustainable cities and society*, 48:101544, 2019.

[11] Thilo Wellmann, Franz Schug, Dagmar Haase, Dirk Pflugmacher, and Sebastian van der Linden. Green growth? on the relation between population density, land use and vegetation cover fractions in a city using a 30-years landsat time series. *Landscape and Urban Planning*, 202:103857, 2020.

[12] Jing Zhong, Jiafeng Liu, Limin Jiao, Xihong Lian, Zhibang Xu, and Zhengzi Zhou. Assessing the comprehensive impacts of different urbanization process on vegetation net primary

productivity in wuhan, china, from 1990 to 2020. *Sustainable Cities and Society*, 75:103295, 2021.
