# Supplementary material for: Performance evaluation of inclusive green growth in China: Dynamic evolution, regional differences, and spatial correlation
Source: PLoS One. 2024 Jul 25;19(7):e0305338. doi: 10.1371/journal.pone.0305338 (PMC11271904; doi:10.1371/journal.pone.0305338)
Supplement: Attachment — Submitted filename: Comments on PONE.docx [file pone.0305338.s002.docx]

Comments on PONE-D-24-03913

Inclusive green growth is an essential approach to achieving sustainable development. This manuscript constructs a system of IGGPLs indicators from using city panel data, further using the methods of vertical and horizontal scatter degree and linear synthesis. Spatial correlation models, regional difference decomposition models, and spatial state transition models are employed to investigate the spatial distribution characteristics and dynamic evolution trends of IGGPLs.

Of particular interest in this manuscript is the construction of an evaluation indicator system for IGGPLs from the city level, and the measurement and evaluation of IGGPLs in China's 271 cities, which is a relatively novel perspective, as most studies have focused on the macro-administrative scale of provinces. In contrast, further refinement of the evaluation index system can provide specific guidance for the green transformation of the economy.

But before accepting publication, there are still some elements that need further explanation， which will be more conducive to improving the quality of the manuscript:

(1) The introduction section does not adequately clarify the scientific issues. It is not stated why IGGPLs are analysed from a spatial perspective and it is recommended that the authors add this section.

(2) The *W* matrix used by the authors is the 01 neighbourhood matrix. Would the authors please give full consideration to whether this matrix is sufficiently interpretable and whether a geographic distance matrix, an economic distance matrix, or an economic-geographic nested matrix would be more applicable?

(3) In the results and analyses section, the authors provide a lot of descriptions around the empirical results, but the reasons for the results are not fully elaborated. It is recommended that the authors not only present the empirical results, but also analyse more around the real situation, which may be conducive to a more focused opinion.

In summary, I suggest that the author make minor revisions to the manuscript.
